# HIV-1 virological synapse formation enhances infection spread by dysregulating Aurora Kinase B

**DOI:** 10.1371/journal.ppat.1011492

**Published:** 2023-07-17

**Authors:** James W. Bruce, Eunju Park, Chris Magnano, Mark Horswill, Alicia Richards, Gregory Potts, Alexander Hebert, Nafisah Islam, Joshua J. Coon, Anthony Gitter, Nathan Sherer, Paul Ahlquist

**Affiliations:** 1 John and Jeanne Rowe Center for Research in Virology, Morgridge Institute for Research, Madison, Wisconsin, United States of America; 2 McArdle Laboratory for Cancer Research, University of Wisconsin–Madison, Madison, Wisconsin, United States of America; 3 Institute for Molecular Virology, University of Wisconsin–Madison, Madison, Wisconsin, United States of America; 4 Department of Computer Sciences, University of Wisconsin–Madison, Madison, Wisconsin, United States of America; 5 Department of Chemistry, University of Wisconsin–Madison, Madison, Wisconsin, United States of America; 6 Department of Biomolecular Chemistry, University of Wisconsin–Madison, Madison, Wisconsin, United States of America; 7 Morgridge Institute for Research, Madison, Wisconsin, United States of America; 8 Department of Biostatistics and Medical Informatics, University of Wisconsin–Madison, Madison, Wisconsin, United States of America; University of North Carolina at Chapel Hill, UNITED STATES

## Abstract

HIV-1 spreads efficiently through direct cell-to-cell transmission at virological synapses (VSs) formed by interactions between HIV-1 envelope proteins (Env) on the surface of infected cells and CD4 receptors on uninfected target cells. Env-CD4 interactions bring the infected and uninfected cellular membranes into close proximity and induce transport of viral and cellular factors to the VS for efficient virion assembly and HIV-1 transmission. Using novel, cell-specific stable isotope labeling and quantitative mass spectrometric proteomics, we identified extensive changes in the levels and phosphorylation states of proteins in HIV-1 infected producer cells upon mixing with CD4+ target cells under conditions inducing VS formation. These coculture-induced alterations involved multiple cellular pathways including transcription, TCR signaling and, unexpectedly, cell cycle regulation, and were dominated by Env-dependent responses. We confirmed the proteomic results using inhibitors targeting regulatory kinases and phosphatases in selected pathways identified by our proteomic analysis. Strikingly, inhibiting the key mitotic regulator Aurora kinase B (AURKB) in HIV-1 infected cells significantly increased HIV activity in cell-to-cell fusion and transmission but had little effect on cell-free infection. Consistent with this, we found that AURKB regulates the fusogenic activity of HIV-1 Env. In the Jurkat T cell line and primary T cells, HIV-1 Env:CD4 interaction also dramatically induced cell cycle-independent AURKB relocalization to the centromere, and this signaling required the long (150 aa) cytoplasmic C-terminal domain (CTD) of Env. These results imply that cytoplasmic/plasma membrane AURKB restricts HIV-1 envelope fusion, and that this restriction is overcome by Env CTD-induced AURKB relocalization. Taken together, our data reveal a new signaling pathway regulating HIV-1 cell-to-cell transmission and potential new avenues for therapeutic intervention through targeting the Env CTD and AURKB activity.

## Author summary

HIV-1 spreads efficiently through virological synapses (VSs), cell-cell interfaces induced by interaction of HIV-1 envelope protein on infected cells and the CD4 receptor on uninfected target cells. Using mass spectrometry, we identified changes in the levels and phosphorylation of many cellular proteins in HIV-1-infected cells after co-culture with CD4+ cells. We identified alterations in numerous cellular pathways, including striking effects on genes regulating the cell cycle. Notably, the key cell cycle regulatory kinase Aurora kinase B (AURKB) showed a dramatic decrease in kinase activity. We found that AURKB specifically inhibits HIV-1 Env-mediated cell fusion and cell-to-cell transmission. This suppression is specific to HIV-1 Env and alters Env’s membrane fusion activity. We found that, in T cell lines and primary T cells, HIV-1 envelope-CD4 engagement prevents this suppression by causing premature relocalization of AURKB to nuclear centrosomes, but does not alter the cell cycle state of the infected cell. Thus, we identified cellular signaling pathways altered by co-culturing HIV-1+ and CD4+ T cells, novel effects of HIV-1 on cell cycle regulatory kinases and a previously unknown role for AURKB at the plasma membrane. The results have significant implications for understanding HIV-1 and cell processes and for controlling HIV-1.

## Introduction

Human immunodeficiency virus type 1 (HIV-1) efficiently spreads at direct contacts between infected and uninfected cells, through a structure known as the virological synapse (VS). VS formation is initiated when HIV-1 envelope protein (Env) on the surface of an infected cell interacts with its receptor CD4 on an uninfected cell [1–4]. In cultured cells, spread by cell-cell transfer between T cells is 100–1,000-fold more efficient than cell-free infection through the extracellular space [5–9]. Increasing evidence shows that direct cell-cell spread confers multiple replicative advantages to HIV-1 and may allow HIV-1 to evade aspects of the humoral immune response, innate antiviral restriction factors such as tetherin, and antiretroviral drugs [8,10–13]. VS formation promotes infection of the target cell by focusing virion assembly and release at sites of cell-cell contact, leading to efficient HIV-1 integration and high viral gene expression in the target cell [14–18]. These studies imply that Env:CD4 interaction initiates intracellular signaling to induce, activate, or recruit viral and cellular factors required for VS formation, stabilization and expansion necessary for efficient HIV-1 transmission. However, the host determinants of these processes remain poorly understood.

VS formation and function are potentially valuable anti-viral targets in addition to current highly active antiretroviral therapies (HAART) directed against HIV-1 proteins mediating virus entry, reverse transcription, provirus integration and post-release virion maturation. Such current combination therapy reduces viral load below detection and delays disease progression [19–22], but is problematic due to long-term toxicity, potential development of resistance, and inability to target persistent viral reservoirs. In addition, viral spread in the body through VSs [5,6,8,9] is less susceptible to the humoral immune response and antiviral strategies targeting viral entry. As well as VS-relevant viral functions, important therapeutic targets could include host cell signaling pathways thought to be activated in infected producer cells or uninfected target cells by their contact [23–26]. However, one major challenge to systematic analysis of cell-specific signaling networks after contact of HIV-1-infected cells with uninfected cells is that the distinct signaling responses of the infected and target cells are mixed when the combined cell populations are processed for biochemical assays. To address this challenge, we developed cell-specific protein labeling approaches combining stable isotope labeling of amino acids in cell culture (SILAC) with tandem mass tag (TMT) labeling of peptide pools after isolation [27,28]. The resulting combinatorial [29] labeling allows quantitative mass spectrometry-based phosphoproteomics to identify cell-specific changes in the levels or states of proteins over time in mixed T cell populations. SILAC distinguishes producer and target cells, while TMT labeling allows combining samples in multiplexes that are analyzed by LC-MS/MS as a single pool, providing accurate quantitative comparisons between all conditions within a single multiplex, since the mixed samples are subjected to the same treatments, including any loss or enrichment of specific peptides. Using these approaches, we successfully distinguished proteomic and pathway changes specifically induced by cell-cell contact in the HIV-1-infected cells rather than the uninfected target cells.

Interestingly, three of the proteins most strongly implicated by this proteomic analysis were LCK, WEE1 and Aurora B, kinases regulating T cell proliferation (LCK) [30] or cell cycle (WEE1 and AURKB) [31,32]. Inhibiting LCK and WEE1 reduced cell to cell spread, potentially consistent with certain prior indications for their interaction with HIV-1 [33,34]. In contrast, inhibiting AURKB increased HIV-1 spread, in association with longer lived, extended cell-cell contacts and increased Env fusion activity. AURKB is a component of the chromosome passenger complex (CPC) and a key mitotic regulator essential for chromosome alignment, segregation, cytokinesis and membrane scission [35]. AURKB remains relatively constant in level throughout the cell cycle and is functionally regulated by its subcellular localization [32]. We show that HIV-1 Env:CD4 engagement results in AURKB relocalizing to the centromere in the Jurkat T cell line and primary T cells. This relocalization requires the Env cytoplasmic tail domain (CTD) implicated in cellular signaling [36]. These results imply that AURKB has previously uncharacterized cytoplasmic/plasma membrane function(s) that reduce HIV-1 Env fusion activity, which HIV-1 overcomes by signaling through the Env CTD to transfer AURKB into the nucleus.

## Materials and methods

### Cell lines and cell culture

Jurkat clone E6-1 (American Type Culture Collection (ATCC)) and SupT1 (NIH AIDS Reagent Program, Division of AIDS, NIAID, NIH) T cells were cultured in RPMI-1640 (HyClone, Logan, UT) supplemented with 10% fetal bovine serum and 1% penicillin-streptomycin. HeLa, human embryonic kidney (HEK) 293T and U2OS cells were obtained from ATCC and cultured in Dulbecco modified Eagle medium (DMEM; Gibco) supplemented with 10% bovine calf serum (BCS) and 1% penicillin-streptomycin. HeLa TZM-bl cells were obtained from the NIH AIDS Reagent Program and cultured in DMEM (Gibco) supplemented with 10% BCS and 1% penicillin-streptomycin. HeLa cells stably expressing CD4-YFP were previously described and cultured in DMEM supplemented with 10% BCS, 1% penicillin-streptomycin and 1 μg/ml puromycin. [37] Primary T cells were purchased from ZenBio (Durham, NC) and cultured in RPMI-1640 (HyClone, Logan, UT) supplemented with 10% fetal bovine serum, 1% penicillin-streptomycin and 40 U/ml IL2 (SantaCruz Bio, Santa Cruz, CA).

### Plasmids and chemical inhibitors

A proviral plasmid pNL4-3.GFP-nLuc.R-E- was generated by replacing the *luciferase* coding region in pNL4-3.luc.R-E- (obtained through the NIH AIDS Reagent Program, Division of AIDS, NIAID, NIH, from Dr. Nathaniel Landau) [38] with GFP-fused Nano luciferase (Promega) [39]. Plasmid pSVIII-92HT599.24 encodes a CXCR4 tropic HIV-1 Env protein and was obtained from the NIH AIDS Reagent Program, Division of AIDS, NIAID, NIH; a gift of Dr. Beatrice Hahn [40]. Full length and ΔCTD NL43 and SF162 envelope proteins were cloned from viral genomic DNA obtained from the NIH AIDS Reagent Program, Division of AIDS, NIAID, NIH under control of the viral LTR as previously described [37]. A Rev-dependent, mCherry-fused HIV-1 Gag expression plasmid, pGag-mCherry has been previously described [37]. HIV-1 visible viruses were engineered into the NL4-3 strain with *Env*, *nef* and *vpr* deletions and engineered to encode Gag fused fluorescent proteins [3,41], [38]. *CFP*, *YFP*, *mScarlet* and *iRFP670* transgenes were inserted in frame, flanked by protease sites, between Gag sequences encoding Matrix (MA) and Capsid (CA) subunits, using standard cloning techniques. A HIV-1 Rev expression plasmid, pLP2 was obtained from Invitrogen (K497500, ViraPower Lentiviral Packaging Mix). The following expression plasmids encoding fluorescent cellular proteins were obtained from Addgene: GFP-kinase dead AURKB (108492), mEmerald-kinase dead AURKA (54010), SOGO-GFP (108494), mCherry-INCENP(108487), mCherry-CENPB (55016), mAzurite-Histone 2B (55232), pLenti-Blast-PIP-FUCCI (138715). In some instances, the fluorescent protein tags were changed using traditional cloning methods. All clones were confirmed by Sanger sequencing. Inhibitors used in this study were diluted in DMSO and are listed in Table 1.

**Table 1:** Kinase and phosphatase inhibitors used in spread assay.

Target^1^	Inhibitors^2^	CAS number^3^	Source^4^	Stock (mM)^5^	concentration range (mM)^6^	Effect on cell to cell spread^7^	Effect on cell viability^8^
AURKA	TCS7010	1158838-45-9	Tocris Bioscience	20	0.16–20	none	none
AURKB	Barasertib (AZD1152-HQPA)	722544-51-6	SelleckChem	20	0.1–100	Increased (Fig 4)	none
AURKB	Hesperidin	520-26-3	SelleckChem	10	0.8–40	Increased (Fig 5)	none
CDK1	RO-3306	872573-93-8	MilliporeSigma	20	0.16–20	Reduced (Fig 4)	none
CDK9	LDC000067	1073485-20-7	SelleckChem	10	0.16–20	Reduced (Fig 4)	none
CDK13	THZ531	1702809-17-3	SelleckChem	10	0.08–10	Reduced (Fig 4)	none
DYRK1A	Harmine	442-51-3	Abcam	100	25–100	Reduced (Fig 4)	none
EEF2K	A-484954	142557-61-7	MilliporeSigma	10	0.8–100	none	none
LCK	Lck Inhibitor	213743-31-8	EMD Millipore	20	0.1 to 100	Reduced (Fig 4)	None
ULK1	SBI-0206965	1884220-36-3	MilliporeSigma	10	4–500	none	producer cells dead at 100 mM
WEE1	MK1775	955365-80-7	SelleckChem	20	0.1 to 100	Reduced (Fig 4)	None
WNK1	STOCK2S-26016	332922-63-1	R&D Systems	10	0.08–10	none	Producer cells dead at 10mM
DUSP3/ PTPN1	RK-682	150627-37-5	Santa Cruz Biotech.	10	0.8–100	none	none
PTPRC	CD45 Inhibitor VI	345630-40-2	EMD Millipore	10	0.08–10	none	producer cells dead at 10mM
ROCK	Y-27632	129830-38-2	SelleckChem	10	0.8–100	Reduced (Fig 4)	none

^1^cellular kinase or phosphatase target of inhibitor

^2^Supplier name for inhibitor

^3^Chemical abstract services registry number

^4^Name of the company that supplied the chemical

^5^Concentration of the inhibitor after resuspension in DMSO

^6^Concentration range tested in the spread assay

^7^Effect on HIV spread, Figure references provided as appropriate, none indicates < 20% change

^8^Viability effect on either producer or target cells, none indicates < 20% change in either cell type

### Combined stable isotope labeling with amino acids in cell culture (SILAC) and tandem mass tag (TMT)-based phosphoproteomic time course analysis

#### SILAC labeling

Jurkat “light” HIV-1 producer cells were cultured in RPMI-1640 (Gibco) supplemented with 10% dialyzed FBS and 2 mM GlutaMAX (Gibco). SupT1 “heavy” uninfected target cells were cultured in RPMI-1640 (Gibco) supplemented with 10% dialyzed FBS (Gibco) and 2 mM GlutaMAX (Gibco), with L-lysine and L-arginine replaced by [^13^C_6_, ^15^N_2_]-L-lysine and [^13^C_6_^1^]-L-arginine. To prevent arginine-to-proline conversion in “heavy” labeled SupT1 cells, 100 mg/L of additional L-proline was added to the “heavy” SILAC medium. Low passage Jurkat and SupT1 cells were washed and grown as described above for eight doublings, with label incorporation confirmed by MS.

#### Co-culture of HIV-1 producer and uninfected target T cells

To obtain a sufficient number of HIV producer cells for mass spectrometry, pools of 5x10^6^ “light” Jurkat cells were electroporated with 5 μg of the HIV genome plasmid pNL4-3.GFP-luc.R-E- and 3 μg of the Env expression plasmid pSVIII-92HT599.24 using the Neon Transfection System (Invitrogen, Carlsbad, CA). 48 hours post transfection, transfected cells were combined, washed with PBS and resuspended in light SILAC culture medium. An equivalent number of “heavy” SupT1 target cells were mixed with “light” Jurkat HIV-1 producer cells and incubated at 37°C for 0, 5 or 60 min prior to adding ice-cold PBS containing protease and phosphatase inhibitor cocktails (MilliporeSigma, Burlington, MA) directly to the cells, centrifuging at 300XG, 5 min at 4°C, flash freezing in liquid nitrogen and storing at -80°C. For experiment 1, for each of the 10 samples comprising multiplex 1, 2x10^6^ cells from each cell population were co-cultured. For experiment 2, for each of the 20 samples comprising multiplexes 2 and 3, 8x10^6^ cells from each cell population were co-cultured.

#### Lysis and digestion

Frozen cell pellets were thawed on ice, homogenized and lysed in 8 m urea, 50 mm Tris (pH 8.0) supplemented with PhosSTOP phosphatase inhibitors (Roche, San Jose, CA, USA), and Complete Mini EDTA-Free Protease Inhibitor Cocktail (Roche). Protein concentrations were estimated using the bicinchoninic acid assay (Thermo Fisher Scientific, Rockford, IL, USA). To reduce and alkylate, 10 mm tris(2-carboxyethyl)phosphine (TCEP) and 40 mm chloroacetamide were added to each sample. Urea was diluted to 1 M with 50 mM tris pH 8 and samples were digested overnight with trypsin at a protein to enzyme ratio of 50:1. Samples were desalted, and peptide content was measured using the peptide colorimetric assay (Thermo Fisher Scientific, Rockford, IL, USA). From each sample, a 300 μg aliquot was labeled with tandem mass tag (TMT) and pooled for analysis [28,42].

#### Phosphorylation enrichment and fractionation

Phosphopeptides were enriched using previously reported methods [43]. In brief, peptides were mixed with magnetic Ti-IMAC(IV) beads (ReSyn Biosciences, Edenvale, Gauteng, South Africa) in 80% acetonitrile/6% TFA (binding buffer) for 20 minutes and washed 3 times with binding buffer and once with 80% acetonitrile. Phosphopeptides were eluted with 1% ammonium hydroxide in 50% acetonitrile. The resultant phosphopeptide sample and flow-through phospho-depleted peptide sample (peptide) were each fractionated using high pH reversed phase HPLC separation to produce 12 to 16 total phosphopeptide and peptide fractions. Each fraction was dried using a vacuum centrifuge and resuspended in MS-grade water with 0.2% formic acid for subsequent mass spectrometry analysis.

#### LC-MS/MS

For the first experiment, including the 10 multiplex 1 samples, each fraction was loaded onto a 75 μm internal diameter, capillary column with an embedded electrospray emitter and packed with 30 cm of BEH C18, 1.7 μm particles (Waters, Medford, MA, USA) [44]. Peptides were separated over 90 min by nano-liquid chromatography using an Ultimate 3000 RSLC (Thermo Fisher Scientific, San Jose, CA, USA). Samples were loaded suspended in 100% Solution A (0.2% formic acid) with Solution B (70% acetonitrile, 0.2% formic acid) ramped to 50% over the course of the separation, followed by washing with 100% Solution B and finally a re-equilibration in 100% Solution A. Experiment 1 peptides were analyzed using an Orbitrap Fusion MS using a 3 second cycle time method and 60,000 resolving power survey scan, followed by tandem mass spectrometry (MS/MS) scans also collected at 60,000 resolving power. Peptides were fragmented by 0.7 m/z isolation with the quadrupole followed by higher energy collisional dissociation (HCD) at 35% normalized collision energy. All fractions were analyzed using automatic gain control (AGC) targets of 1 × 10^6^ and 1 × 10^5^ for MS and MS/MS scans, respectively. MS maximum injection times for all fractions were set at 100 msec for survey scans and 75 msec for MS/MS. Phosphopeptide fractions were analyzed using MS/MS maximum injection times set at 120. Only peptides with charge states from +2 to +8 were selected for MS/MS using an exclusion duration of 20 s. Each phosphopeptide fraction was run in duplicate. In experiment 2, the samples of multiplexes 2 and 3 were analyzed similarly, except that the samples were analyzed using a 120 min method with mass analysis for these samples performed on an Orbitrap Elite system using a top 20 method with 30,000 MS/MS resolving power, 2 m/z isolation, 45 second dynamic exclusion, and 250 msec maximum injection time.

#### Data analysis

The OMSSA algorithm and COMPASS software suite were used for searching and processing data, respectively [45]. Raw files, available at https://chorusproject.org under project #1785, were first converted to text files and scored against theoretical spectra from a target-decoy species-specific reference proteome database, downloaded from UniProt, using the OMSSA search engine. Tryptic peptides were searched with one or three missed cleavages, for peptide and phosphopeptide samples, respectively. Cysteine carbamidomethylation, and TMT modification of the peptide N-terminus and lysine residues were set as fixed modifications. Methionine oxidation was set as a variable modification for all samples. Phospho-enriched samples were also searched for phosphorylation with neutral loss on serine and threonine as well as phosphorylation of tyrosine. All searches were performed using 25 ppm tolerance around the monoisotopic precursor mass and 0.01 Da tolerance on fragment ion masses. The COMPASS software suite was used to filter search results to achieve a 1% unique peptide false discovery rate (FDR; based on E-value and ppm mass error). Protein identifications were grouped based on the rules of parsimony and filtered to 1% FDR. Localization of phosphorylation sites was performed with an adopted phosphoRS algorithm [46]. Only sites with ≥ 75% localization probability were reported.

### Computational analysis to identify proteins and networks enriched in HIV-1 producer cells after co-culture with uninfected cells

#### Normalization and fold change calculation

Fold changes were calculated with Python (v3.9.7). Any duplicate mappings were replaced with the mean of the duplicates. Each TMT multiplex was quantile normalized, then means were taken across replicates for each time point. For the primary WT HIV analyses, only WT conditions from each multiplex were used for quantile normalization. For the control analyses, all conditions on the multiplex were used for quantile normalization. Fold changes at 5 and 60 min were both calculated with respect to 0 min, comparing only replicates within multiplexes. Volcano plots were created with the Python packages matplotlib (v3.6.2), numpy (v1.21.2), pandas (v1.3.4), sanbomics (v0.0.7), and seaborn (v0.12.1). [47–50].

#### Significance testing and filtering

Significance calculations were performed using the limma package (v3.50.3) and the q-value package (v2.26.0) [51,52] with R (v4.1.1). Proteins and phosphopeptides were considered significantly different between time points if they had a q-value ≤ 0.1 and a fold change ≥ 1.5 in magnitude. These cutoffs were chosen to be inclusive because subsequent network analysis would further filter out proteins that were isolated in a network context.

#### Prize-collecting Steiner forest network analysis

We performed network enrichment using the Prize-collecting Steiner forest (PCSF) algorithm. PCSF creates subnetworks from sets of proteins and a background protein-protein interaction network by finding connections between proteins of interest. In PCSF, proteins of interest are given prizes, and all edges in the reference set of protein-protein interactions are given costs. The objective is to select a subnetwork from the larger background network that maximizes the prizes of the selected proteins and minimizes the costs of the selected edges. The subnetwork is a forest-structured graph *F = (V*_*F*_, *E*_*F*_*)* that optimizes the following function:

$$\underset{F}{argmin}\sum_{v\notin V_{F}}\left(\beta \cdot p(v)-\mu \cdot d(v)\right)+\sum_{e\in E_{F}}c(e)+\omega \cdot \kappa$$

where *p(v)* is the positive prize on each protein vertex, *c(e)* is the positive cost on each edge, *d(v)* is the degree of each vertex, and *κ* is the number of trees (connected components) in the subnetwork. The parameters *β*, *μ*, and *ω* are used to control the desired properties of the subnetwork such as the size. Choices of prizes, costs, and parameters are explained below. We used the Omics Integrator [53] implementation of PCSF, which solves PCSF via a message passing algorithm [54].

#### Network analysis inputs

All significant proteins and phosphopeptides were used to generate protein prizes for PCSF. The magnitude of each protein’s log transformed q-value was used as its prize, taking the maximum value if both the protein abundance and phosphorylation changes were significant. Different prizes were created for the 0 to 5 min and for 0 to 60 min comparisons. The protein interaction network combined general protein-protein interactions from iRefIndex (v13.0) [55] and kinase-substrate interactions from PhosphoSitePlus using the edge scores from Köksal et al. [56,57]. We ran PCSF such that each tree started with one of the interaction partners of HIV-1 Env listed in the NCBI HIV database (https://www.ncbi.nlm.nih.gov/genome/viruses/retroviruses/hiv-1/interactions/browse/). We selected all interaction partners listed as “binds” or “interacts with”, totaling 462 proteins.

#### Parameters and computation

We ran PCSF in a grid search with many different parameter combinations, analyzing the 5 and 60 min prizes separately. *β* was tested from 0 to 5 in increments of 0.5. *ω* was tested from 0 to 1.5 in increments of 0.1 and from 1.5 to 3 in increments of 0.5. *μ* was tested from 0 to 0.9 in increments of 0.015. Calculations were run in parallel with HTCondor [58].

#### Network filtering and aggregation

After generating subnetworks for each parameter combination, we discarded: any empty networks; networks that did not have a connected component with at least 25 vertices; networks in which one vertex was connected to 10% or more of the total vertices (which indicates a dominant hub node); and networks that contained a chain of more than 8 vertices without any nonzero prizes. An additional filter for networks whose vertices were not at least half prize nodes did not affect any of the networks. We then took the union of all remaining networks to create a final combined network for each time point, 5 and 60 min. We considered the confidence of a vertex or edge in the combined network to be the percent of original networks in which that vertex or edge appeared. For instance, the 60 min 30% confidence subnetwork is the collection of all edges and vertices that appeared in at least 30% of the 60 min filtered networks. Subnetwork visualizations were created with Cytoscape [59].

#### Gene Ontology enrichment

We analyzed all of the proteins in the combined 5 and 60 min PCSF networks with the Database for Annotation Visualization and Integrated Discovery (DAVID) [60] using their official gene symbols. The Functional Annotation Tool in DAVID (v6.8) was run using the default parameters and Gene Ontology (GO) biological process terms. All GO terms that met the inclusion criteria were downloaded as Functional Charts. GO terms with a maximum Benjamini-Hochberg adjusted p-value of 0.001 were considered significant.

#### PhosFate Profiler kinase enrichment analysis

We estimated kinase activities from the phosphorylation log2 fold changes at 5 and 60 min using PhosFate Profiler [61]. PhosFate Profiler computes an enrichment score for kinases using the Kolmogorov–Smirnov statistical test in a similar fashion to gene set enrichment analysis. We appended the log2 fold changes from both 5 min phosphoproteomic experiments and, separately, both 60 min experiments. If there were multiple phosphorylated sequence indices reported for an isoform, we split them to appear one per line with the same log2 fold change.

### HIV-1 cell-cell transfer assay

5x10^6^ Jurkat T cells were electroporated with 5 μg of the HIV-1 genome plasmid pNL4-3.GFP-luc.R-E- and 3 μg of the HIV-1 Env expression plasmid pSVIII-92HT599.24 using the NeonTransfection System (Invitrogen, Carlsbad, CA). After 24 hours, Jurkat HIV-1 producer cells were washed with PBS, treated with inhibitor (Table 1) or vehicle control, and incubated for 2 hours at 37°C. Subsequently, 2x10^4^ HeLa TZM-bl target cells, expressing HIV-1 TAT-inducible firefly luciferase, were mixed with 2x10^4^ inhibitor-treated Jurkat HIV-1 producer cells in a 96-well plate format and incubated at 37°C. After 20 min or 2 hours, Jurkat HIV-1 producer cell suspension was removed, and HeLa TZM-bl target cells were washed with PBS. Complete culture medium was added back to HeLa TZM-bl target cells, and the cells were incubated at 37°C. After 48 hours, cell viability was determined using the CellTiter-Glo assay (Promega, Madison, WI), and TAT-driven luciferase expression was measured using BriteLite Plus reagent (Perkin Elmer, Boston, MA) according to the manufacturer’s instructions. Luciferase activity was read using a Victor X5 Multilabel Plate Reader (Perkin Elmer, Boston, MA). The % of virus cell-cell transfer was calculated by normalizing measured luciferase activity to the no inhibitor treatment control plotted against inhibitor concentration, using GraphPad Prism v7.0 (GraphPad Software, Inc., San Diego, CA).

### HIV-1 content mixing/syncytia assay

5x10^6^ Jurkat T cells were electroporated with 2 μg pNL4-HIV-TAT expression vector [62] and 3 μg of Envelope expression plasmid pSVIII-92HT599.24, with 1 μg GFP expression plasmid (pEGFP-N1, Clonetech) to monitor transfection efficiency and 2 μg carrier DNA (pBluescript, Stratagene) using the NeonTransfection System (Invitrogen, Carlsbad, CA). After 24 hours, Jurkat HIV-1 producer cells were washed with PBS, treated with inhibitor or vehicle control, and incubated for 2 hours at 37°C. The Jurkat cells were pelleted, washed with PBS and resuspended in fresh media and 2x10^4^ inhibitor-treated Jurkat HIV-1 producer were to 2x10^4^ HeLa TZM-bl target cells, expressing HIV-1 TAT-inducible firefly luciferase, in a 96-well plate format and co-cultured at 37°C for 24 hours. 24 hours after co-culture cell viability and TAT driven luciferase activity was assayed as described above.

### HIV-1 cell-free infection assay

To generate HIV-1 virions, 5x10^6^ Jurkat T cells were electroporated with 5 μg of the HIV-1 genome plasmid pNL4-3.GFP-luc.R-E- and 3 μg of the HIV-1 Env expression plasmid pSVIII-92HT599.24 using the NeonTransfection System (Invitrogen, Carlsbad, CA). After 24 hours, HIV-1-expressing producer cells were pelleted and washed to remove extracellular virions. The cells were resuspended in media containing kinase inhibitor and incubated for an addition 2hr. Treated cells were then pelleted and the virion-containing supernatant mixed with TZM-bl reporter cells, diluting the inhibitor 5-fold, and then incubated for 48 hrs to allow for infection and reporter gene expression. Supernatants were then removed, and luciferase expression was measured using the BriteLite Luciferase Assay Reagent (Promega, Madison, WI) as described above. The % of virus infectivity was calculated by normalizing to the measured luciferase activity of the no inhibitor treatment control, plotted using GraphPad Prism v7.0 (GraphPad Software, Inc., San Diego, CA).

### Western blotting

5x10^6^ Jurkat T cells were electroporated with 5 μg of the HIV-1 genome plasmid pNL4-3.GFP-luc.R-E- and 3 μg of the HIV-1 Env expression plasmid pSVIII-92HT599.24 using the NeonTransfection System (Invitrogen, Carlsbad, CA). After 24 hours, Jurkat HIV-1 producer cells were washed with PBS. 2x10^6^ Jurkat HIV-1 producer cells were then treated with inhibitor and incubated at 37°C. After 18 hours, to harvest released virions, supernatant was collected, filtered, underlaid with 30% (wt/vol) sucrose in PBS and subjected to centrifugation at >21,000 xg for 2 hours at 4°C, with virion pellets resuspended in 50 μl dissociation buffer (8M urea, 5% sodium dodecyl sulfate [SDS], 40 mM Tris-HCl pH 7, 0.1 mM EDTA, 25% glycerol, 4% β-mercaptoethanol, bromophenal blue). Cell lysates were also harvested, with cells washed with ice-cold PBS, resuspended in 200 μl dissociation buffer containing Protease and Phosphatase Inhibitor Cocktail (MiliporeSigma, Burlington, MA) and homogenized using Omega Homogenizer Columns (Omega Bio-Tek, Norcross, GA). Proteins were boiled and resolved using SDS-polyacrylamide gel electrophoresis (PAGE) followed by transfer to PVDF membranes and blotting using antibodies against the gp120 subunit of HIV-1 Env (ab21179, 1:1000; Abcam), the p24 Capsid (p24CA) subunit of HIV-1 Gag (sc-69728, 1:250; Santa Cruz Biotechnology), phospho-AKT S473 (4060, 1:1000; Cell Signaling Technology), phospho-p38 (MAPK14)T180/Y182 (sc-17852-R, 1:250; Santa Cruz Biotechnology), phospho-CDK1 Y15 (9111, 1:1000; Cell Signaling Technology), phospho-Aurora A T288/Aurora B T232/Aurora C T198 (2914, 1:1000; Cell Signaling Technology) and actin (sc-1616, 1:250; Santa Cruz Biotechnology) prior to incubation with corresponding anti-goat, anti-rabbit or anti-mouse secondary antibodies conjugated to infrared fluorophores IRDye800 or IRDye680 (LiCor Biosciences, Lincoln, NE). Fluorescent signal was detected using an Odyssey CLx infrared imager (LiCor Biosciences, Lincoln, NE).

### Confocal microscopy

To monitor HIV-1 trafficking to the VS using microscopy, we transfected previously validated HIV-1 NL4-3 visible viruses with *Env*, *nef* and *vpr* deletions engineered to encode Gag fused fluorescent proteins (Gag-CFP, Gag-YFP, Gag-iScarlet or Gag-iRFP) [38] along with plasmids encoding fluorescent tagged AURKB and centromeric proteins described above. For imaging, 5x10^6^ Jurkat T cells were electroporated in the presence of a total of 10 μg plasmid DNA containing 5 μg of plasmid encoding HIV visible virus, 3 μg of the HIV-1 Env expression plasmid pSVIII-92HT599.24 or other envelope expressing protein described above, and 2 μg fluorescent cellular protein or carrier plasmid (pBluescript, Stratagene) using the NeonTransfection System (Invitrogen, Carlsbad, CA). After 24 hours, Jurkat HIV-1 producer cells were treated with inhibitors as described above, washed, mixed with an equivalent number of target SupT1 cells for 20 min on a poly-D-lysine slide (Neuvitro) before fixing in 4% paraformaldehyde for 10 min and mounting. Primary T cells were transfected as Jurkat cells but outgrowth was done in media containing 40 U/ml IL2. Alternatively, to assess the effects of VS formation on cellular protein distribution, Jurkat producer cells were added for 20 min to a monolayer of Hela or U2OS cells engineered to express CD4, CD4-YFP or CD4-CFP plated in 12-well chamber slides (Ibidi) as previously described [37]. Cells were fixed in 4% formaldehyde and mounted in Molwiol containing DABCO preservative. Confocal imaging was performed on a Nikon A1R (Nikon Corporation) inverted confocal microscope using a 60X (N.A. 1.4; Plan Apo) oil immersion objective (Nikon Corporation) using Nikon NIS Elements software (version 4.20.03). Images were processed and analyzed using FIJI/ImageJ2 [63].

### Live cell imaging

5x10^6^ Jurkat T cells were electroporated in the presence of a total of 10 μg plasmid DNA containing 5 μg of plasmid encoding HIV visible virus, 3 μg of the HIV-1 Env expression plasmid pSVIII-92HT599.24 or another envelope expressing protein described above, and 2 μg fluorescent cellular protein or carrier plasmid (pBluescript, Stratagene) using the NeonTransfection System (Invitrogen, Carlsbad, CA). 24 hours post transfection, cells were treated with inhibitor for 2 hours, washed to remove inhibitor and resuspended in fresh media and mixed at a 1:2 ratio with Hela cells expressing CD4-YFP (4X10^4^) seeded in an 8-well glass-bottom chamber slide (Ibidi) as previously described [37]. Images were collected with a 20X objectives on an Eclipse Ti automated epifluorescence microscope (Nikon). Movies were postprocessed and analyzed using NIS Elements (Nikon) and FIJI/ImageJ2 [63] software packages as previously described [37].

### Cell cycle and AURKB relocalization assay

For imaging, 5x10^6^ Jurkat T cells were electroporated using the NeonTransfection System (Invitrogen) with a total of 10 μg plasmid DNA containing 2 μg of plasmid encoding mAzurite-Histone H2B fusion protein (Addgene, 55232) to mark the transfected cells, and 3 μg of an envelope expressing protein, and 5 μg of PIP-Fucci 2-color fluorescent cell cycle reporter plasmid [64] (Addgene, 138715), which encodes the YFP-PIP protein cell cycle marker that is degraded at the start of S-phase and reaccumulates after S-phase is completed, and mCherry-Geminin marker, which accumulates during S-phase, but is degraded after completion of mitosis. 24 hours post transfection, cells were fixed and stained for endogenous AURKB using mouse-anti-AIM-1 (AURKB) antiserum (BD Bioscience, Cat# 611083), at a 1:2000 dilution, followed by donkey anti-Mouse-Alexafluor 647 secondary antiserum, at a 1:2000 dilution. Cells were mounted in Molwiol containing DABCO and imaged by confocal microscopy as described above. 10 independent 60X fields were captured as above and processed using FIJI/ImageJ2 [63]. Transfected cells were scored for cell cycle stage and AURKB relocalization. At least 150 cells were analyzed per condition for each experiment.

## Results

### Quantitative analysis of coculture-induced phosphoproteomic and proteomic changes during HIV-1 spread between T cells

**Fig 1:**
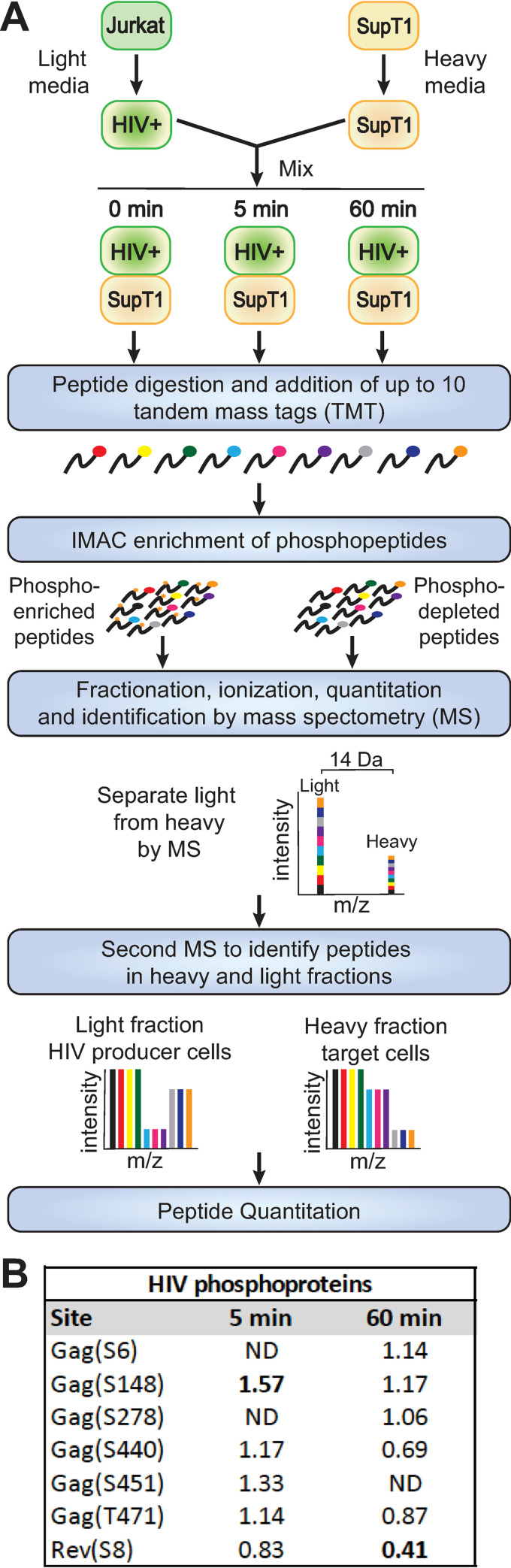
Summary of multiplexed mass spectrometry proteomic and phosphoproteomic analyses. (A) Schematic of experimental design for the phosphoproteomic and proteomic analyses. HIV expressing Jurkat cells grown in light media are mixed with target SupT1 cells grown in heavy media for 0, 5 or 60 minutes, with three or four replicates for each condition. Samples were harvested, peptides digested and the peptides in each sample were ligated to a tandem mass tag (TMT). IMAC chromatography was used to enrich phosphoproteins and liquid chromatography mass spec (LC-MS) was used to resolve the light and heavy samples, and a second MS was run to resolve the TMT. (B) HIV proteins with altered phosphorylations 5 and 60 min after coculture.

To better define the molecular mechanisms underlying HIV-1 spread between T cells, we adapted cell type-specific, metabolic stable isotope labeling of amino acids in cell culture (SILAC) for simultaneous, differential, quantitative mass spectrometry-based proteomics from two independent T cell populations in mixed culture (Fig 1A). We chose Jurkat and SupT1 cells because they are well-characterized T cell models for HIV-1 replication and infection [65,66]. SupT1 cells were chosen as uninfected target cells because they express high levels of CD4, the receptor for HIV-1 Env [67]. Jurkat cells were chosen as producer cells because of their naturally low levels of CD4, which is further downregulated by Vpu [68] after virus expression. Thus the Jurkat producer cells are less reactive targets for Env-mediated cell interaction than the high CD4-expressing SupT1 target cells, promoting strong Env:CD4 contacts between Jurkat:SupT1 cells and reducing Jurkat:Jurkat interactions that could cloud analysis. We selected 5 minutes after mixing to catch rapid phosphorylation responses induced by cell to cell contact and 60 minutes to determine if those changes were long lasting and to see potential additional changes in protein levels. We confirmed that these time points could identify phosphorylation changes by mixing HIV-1-expressing Jurkat cells with naive SupT1 cells and performing western blot analysis for phospho-AKT and phospho-MAPK14 (S1 Fig), which have previously been shown to be altered by HIV-1 synapse formation [26,69–71]. Phosphorylation of AKT was induced after 5 min of mixing and remained elevated at 60 min. In contrast, MAPK14 phosphorylation was unchanged at 5 min and suppressed by 60 minutes post mixing, suggesting that these two timepoints would identify important phosphorylation changes induced by cell to cell contact. Notably, these two control proteins also were identified in our mass spectrometry phosphoproteomics (see below, Table 2), further validating the system.

**Table 2:** Phosphorylation changes after VS formation.

Enzyme^1^	Regulatory sites^2^		
**Kinases**	**5 and 60 min**	**5 min**	**60 min**
AAK1		**S678**	**T389**
AKT1	**S473**,S477		
AKT2	**S474,S478**		
BAZ1B	**S189,S347**		S312,**S349,S947**
BMP2K	**S1107, S1111**		
CDK9	S464	T303,	
CDK11B	S81	S178	
DYRK1A	**S529**		**S758**
EEF2K	S462	**S445**	**S470,S474**
GTF2F1	**T331**	**S433**	
LCK	Y424		
MAP3K2	**S153**		
MAP4K4	**S680**,S886,S889,S934	S549	
MAPK14	**T180**		
MARK3	**T530**		
NEK1	**T344**		
NEK4	S661		
PAK4	**S104**		**S99, S148**
SIK3		T463	S668
TTN	-	-	-
ULK1	S605		
WEE1	**S150**		**T187,T190**
WNK1		**S378**	**T60**
ZAP70	**Y493**		
**Phosphatases**			
DUSP3	-	-	-
INPP5F	S935		
PPM1H	**T113**	**S124**	
PTPN1	**S50,S378**		
PTPN7		S149	S248
PTPN18	T409		
PTPRC		S1009	S975
SSH2		S1286	T1449

^1^Cellular kinases and phophatases with phosphosites significantly modulated (fold change ≥ 1.5 and q-value ≤ 0.1)

^2^Bolded sites were previously identified, unbolded sites were identified in this study. Changes observed at 5, 60, or both are indicated. Dashes (DUSP3) indicates changes in protein level but no change in phosphorylation state.

To differentiate the two cell lines in mass spectrometry, SupT1 target cells were labeled for eight doublings with “heavy” amino acids, with ~100% incorporation of ^13^C arginine and ^13^C^15^N lysine confirmed by mass spectroscopy, while the Jurkat producer cells were grown in normal “light” media (Fig 1A). To further focus analysis on cell contact-induced changes in producer cells and minimize downstream effects on signaling pathways [34] from cell-free infection, we transfected Jurkat producer cells with a plasmid carrying an HIV-1 provirus [38,72] expressing a GFP—NanoLuc luciferase fusion gene (Promega), and an HIV-1 Env and Nef expression plasmid [40]. After 48 hours, when >80% of these transfected cells showed HIV-1-directed GFP expression by fluorescence microscopy, cells were washed to remove any virion-like particles that may have started to bud, and the producer cells were mixed in 1:1 ratio with the HIV-1-negative, “heavy” amino acid-labeled SupT1 target T cells.

**Fig 2:**
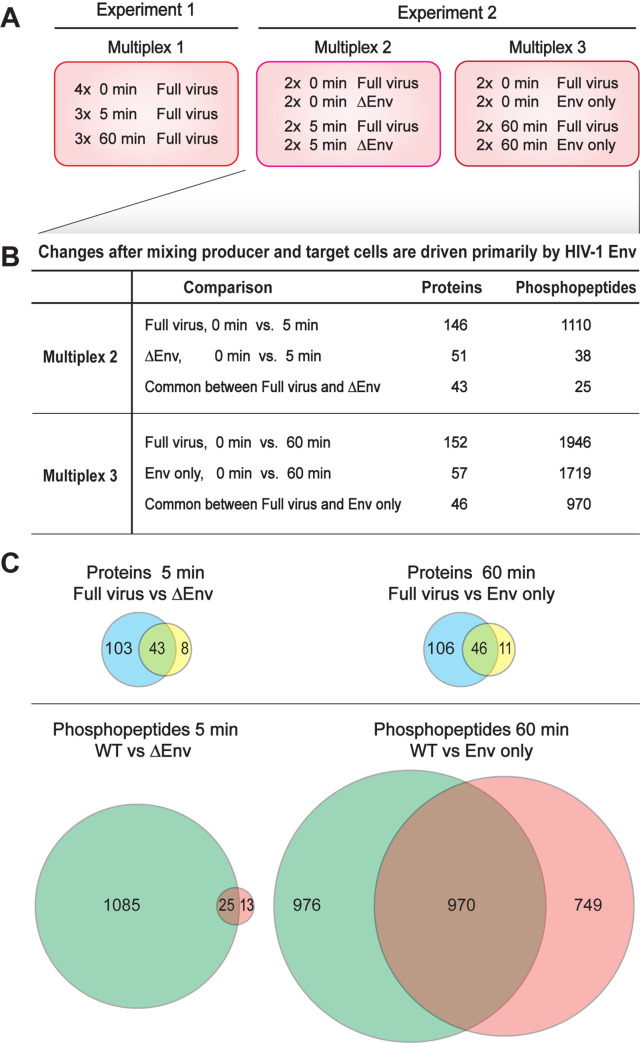
Multiplex analysis used for MS analysis of HIV Env-mediated cell signaling. (A) Schematic showing 3 different multiplexes used in TMT-mass spec analysis. Multiplex 1 contained full HIV expressing producer cells mixed with uninfected target cells for 0 min. (quadruplicate), 5 min. (triplicate) and 60 min (triplicate). Multiplex 2 had duplicates of WT full virus at 0 and 5 min and duplicates of HIV lacking Env (ΔEnv) at 0 and 5 min. Multiplex 3 consisted of WT full virus in duplicate at 0 and 60 min and duplicates of cells only expressing Env (Env only). (B) Tabular comparison of shared total protein and phosphopeptide changes identified with the control samples in multiplexes 2 and 3 are shown. (C) Venn diagrams indicating relative Env dependent changes at 5 and 60 minutes are shown below.

For proteomic analysis, parallel co-cultures were frozen in liquid nitrogen either immediately after mixing (0 min) or after 5- or 60-min incubations at 37°C to allow time for CD4:Env interaction, signaling initiation, cell responses and VS formation (Fig 1A). To improve identification of candidate proteins for further downstream analysis, two independent co-culture experiments were performed, each with two or more replicates of each condition (Fig 2A; see also further description below). After freezing, cells were lysed and proteins digested as described in Materials and Methods. To most accurately compare unique peptide profiles for each experimental condition and time point, total peptides from each independent co-culture sample were labelled using a distinct tandem mass tag (TMT) [28] and then mixed for pooled analysis (Fig 1A). The pooled TMT-labeled samples then were fractionated by immobilized metal affinity chromatography into a flow-through non-phosphopeptide sample and a bound and eluted phosphopeptide-enriched sample, which were analyzed separately by liquid chromatography-tandem mass spectrometry (LC-MS/MS). This combinatorial approach of cell-specific labeling and TMT allows differentiating changes in the proteomes of producer and target cells. Since infection-induced changes in signaling in target cells have already been significantly studied [23,34,73,74], we chose to focus here on co-culture-induced changes in the HIV-1-positive producer Jurkat T cells, which have not been as rigorously examined due to the technical challenges mentioned above. Analysis of parallel changes in the initially HIV-1-negative SupT1 target T cells will be presented elsewhere.

### Coculture has only modest effects on HIV-1 protein levels and phosphorylation

We first examined the effects of coculture on HIV-1 proteins levels and phosphorylation state. As expected, we only detected HIV-1 proteins in the “light” producer cell peptides and none in the “heavy” target cell peptides, confirming that SILAC labeling specificity was maintained after cell mixing. HIV protein levels were stable, showing no significant changes (defined as ≥1.5-fold change relative to the 0-min time point) after either 5 or 60 minutes of cell mixing. While many HIV-1 proteins are phosphorylated (reviewed in [75]), no HIV-1 proteins showed consistent phosphorylation changes after both the 5 and 60 minute incubations. Indeed, the only significant changes were a >1.5-fold increase in Gag S148 phosphorylation at 5 minutes and a >1.5-fold decrease in Rev S8 phosphorylation at 60 minutes (Fig 1B). The roles of Gag S148 in HIV-1 CA function have been extensively examined [76–80], but the increase in phosphorylation after cell-to-cell contact has not previously been noted. Similarly, Rev S8 phosphorylation has been previously implicated in Rev downregulation [81,82] but not associated with VS formation (Fig 1B). These data suggest that transient alterations in previously characterized Gag and Rev phosphorylations are stimulated by Env+:CD4+ cell-cell interaction, but major alterations to viral protein levels are not induced.

### HIV-1+ / CD4+ coculture alters the cellular proteome

In contrast to the HIV proteome, the cellular proteome showed significant changes (defined as ≥1.5-fold change and a q-value ≤0.1 relative to the 0-min time point) in protein levels and phosphorylation state (S2 Fig). Across two independent experiments, we identified 5,933 proteins, of which 386 (7%) exhibited changes in protein levels at 5 or 60 minutes after co-culture (S1 Table). Similarly, we observed 12,782 phosphopeptides, and 3,277 (26%) had altered abundance at 5 or 60 minutes after co-culture (S2 Table). While phosphopeptide profiles are generally significantly more variable than total proteomes, 22% of these phosphopeptides (2,796) were altered in both independent experiments.

To create a statistically robust profile of the protein abundance and phosphorylation changes over time in response to WT HIV, experiment 1 (multiplex 1) included four 0 min time points, three 5 min time points and three 60 min time points (Fig 2A). Analyzing this data showed that there was little variation between technical replicates. We therefore performed a second experiment with fewer replicates per condition and added selected controls to test the role of HIV-1 Env in the protein and phosphorylation changes. This second experiment consisted of two separately pooled multiplexes (Fig 2A). Multiplex 2 contained duplicate samples of Jurkat cells transfected with WT HIV or an HIV variant expressing all HIV proteins except Env (ΔEnv), captured at 0 and 5 minutes after mixing with uninfected SupT1 cells. Multiplex 3 contained duplicate samples of Jurkat cells transfected with WT HIV or transfected to only express HIV Env protein, captured at 0 and 60 min after mixing with uninfected SupT1 cells.

Across these multiplexes, relatively few proteins exhibited changes in protein abundance at either 5 or 60 min (146 and 152, respectively) and about half of these responses were correlated with Env expression (Fig 2B and 2C). In contrast, WT virus expression induced dramatic changes in protein phosphorylation at 5 min (1110 phosphopeptides) that further increased at 60 minutes (1946 phosphopeptides) post mixing. Strikingly, when the Jurkat producer cells were transfected with the same HIV genome with Env deleted, only 2% of the phosphorylation changes seen with WT HIV were observed at 5 minutes after mixing with SupT1 cells (Fig 2). This strong Env-dependence implies that Env-mediated interactions drive signaling after mixing HIV-1 expressing cells with CD4+ target cells. Conversely, transfecting Jurkat cells to express only HIV-1 Env, without other HIV-1 factors, reproduced 50% of the phosphoprotein changes induced by WT HIV. Thus, other viral proteins contribute to signaling changes after cell mixing, but the primary driver of signaling is the HIV-1 Env protein.

### Functional enrichment and network analyses of co-culture-induced proteomic and phosphoproteomic changes

As an initial step in further analyzing the basis of the extensive phosphoproteomic changes identified (Fig 2), and because of the general importance of protein phosphorylation in rapid functional responses, we examined the kinases and phosphatases present among the differentially phosphorylated proteins. 24 kinases and 8 phosphatases showed significant changes in phosphorylation at potential internal regulatory sites at both the 5 and 60 min time points (Table 2). Nineteen enzymes harbored phosphorylation changes at regulatory phosphosites confirmed in prior studies (sites bolded in Table 2), and we identified several novel, phosphorylations (unbolded sites, Table 2). Among the phospho-regulated kinases, NEK1, NEK4, SIK3 and WEE1 function in cell cycle [83–86]; 5 are involved in RNA-related processes, including CDK9 and GTF2F1 in transcription [85,87], CDK11B and DYRK1A in RNA splicing [88,89] and EEF2K in translation [90]; and 2 kinases and 4 phosphatases—DUSP3, LCK, PTPN1, PTPN7, PTPRC and ZAP70—play critical roles in TCR signaling [91].

To fully characterize the interaction of pathways altered by co-culturing our HIV-1+ and CD4+ cells, we next performed network analysis on proteins and phosphopeptides modulated by cell-cell contact (S3 Fig). Each phosphoproteomic and proteomic data set from the two experiments was normalized as described in the Materials and Methods. To provide a relatively inclusive analysis, we used a statistical cutoff of fold change ≥1.5 and q-value ≤0.1 to identify peptides whose phosphorylation and/or abundance were modulated at 5 min or 60 min co-culture (S2 Fig). We then constructed a comprehensive network model describing global signaling and regulatory response changes in HIV-1 producer cells upon co-culture with target cells, using a Prize-Collecting Steiner Forest (PCSF) algorithm to integrate the phosphoproteomic and proteomic analyses [53] (S3 Fig). PCSF identifies protein-protein interactions that connect the significantly modulated proteins through high-confidence paths. It also identifies Steiner nodes, which are proteins that, while not directly implicated by the phosphoproteomic or general proteomic analyses, are needed to provide connecting interactions between proteins identified as modulated by cell-cell contact. We examined many PCSF parameter combinations and implemented post-processing steps to prevent inappropriate proliferation of Steiner nodes. The resulting protein interaction networks connected 1,500 proteins with 3,230 connections after 5 min (S4 Fig) and 1,704 proteins with 2,369 connections after 60 min co-culture (S5 Fig).

**Fig 3:**
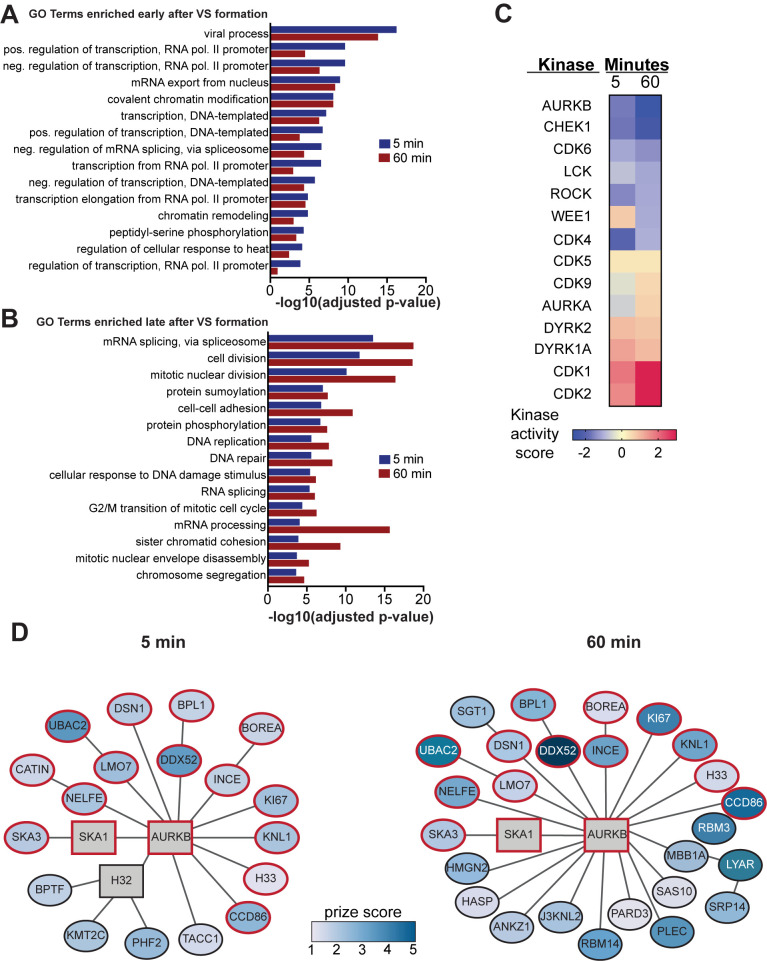
Functional enrichment of protein subnetworks in HIV-1 producer cells after co-culture with uninfected cells. Prize-Collecting Steiner Forest (PCSF) analysis was used to generate subnetworks from proteins with significant changes after 5 or 60 minutes of co-culture. The top 30 enriched Gene Ontology (GO) biological process terms from both 5 min and 60 min subnetworks were divided into those that primarily exhibited (A) early or (B) late enrichment after co-culture. Adjusted p-value indicates the Benjamini-Hochberg corrected p-value. (C) Heat map of the kinase activity score generated by PhosFate Profiler analysis [61], which infers kinase activity from changes in the phosphorylation states of known substrates. Selected cell cycle regulatory kinases and known HIV-1 effectors are shown. A positive score indicates increased phosphorylation while a negative score indicates decreased kinase-specific substrate phosphorylation in HIV-1 producer cells. (D) Selected regions of the protein-protein interaction subnetworks created using the PCSF algorithm from significantly differentiated proteins and phosphopeptides. The subnetworks depict all proteins in the same connected component as AURKB. The 5 min combined subnetwork shows edges with 75% or greater confidence, and the 60 min combined subnetwork shows edges with 90% or greater confidence. Vertex color of the elliptical vertices represents the magnitude of the log-transformed q-values, which were used as protein prizes. Steiner nodes, vertices that were not significantly changed between time points but were included as important connective proteins by the PCSF algorithm, are shown as rectangles. Proteins conserved between 5 and 60 min time points are outlined in red.

To investigate the functions and cellular organization of these dynamic changes, we performed gene enrichment analysis on our 5 min and 60 min PCSF networks using the biological process categories curated by the Gene Ontology (GO) Consortium. The 30 most highly enriched GO terms based on Benjamini-Hochberg adjusted p-value then were sorted based on whether they showed an early response by peaking at 5 min after cell mixing (Fig 3A) or a late response by peaking at 60 min post mixing (Fig 3B). GO terms showing an early enrichment were primarily (12 of 15) involved in gene expression, including positive and negative changes in RNA pol II transcription, nuclear mRNA export, splicing and chromatin remodeling (Fig 3A). While some GO terms related to gene expression, like splicing and mRNA processing, showed a peak enrichment 60 min post cell mixing, the majority of late-enriching GO terms (9 of 15) were associated with cell cycle regulation, including DNA replication, cell division, nuclear envelope disassembly and chromosome segregation (Fig 3B).

### Coculture with CD4+ target cells dramatically modulates the activity of cell cycle regulatory kinases in HIV-1 producer cells

We were particularly interested in how HIV1+ cell interaction with CD4+ target cells induced cellular signaling pathways resulting in late cell cycle changes. To address this, we examined changes in the phosphorylation state and activity of cellular kinases and phosphatases. To identify which kinase activities were affected, we interrogated our protein phosphorylation data using PhosFate Profiler, which infers changes in kinase activity based on quantitative phosphoproteomic data showing changes to known target phosphosites [61]. Positive and negative scores respectively indicated upregulated and downregulated kinase activities (Fig 3C). In total, PhosFate Profiler analysis identified significant regulation of 138 kinases at 5 min of contact and 145 kinases at 60 min of contact (S3 Table).

From all kinases implicated by PhosFate Profiler, we identified 11 that were associated with the cell cycle and that were modulated in activity at both 5 and 60 min after initial cell contact (Fig 3C). For reference, we included three kinases with known effects on HIV-1: CDK9, ROCK, and LCK [34,92–94]. Of these, cell-to-cell contact upregulated the predicted activity of 7 kinases at one or both time points (Fig 3C, bottom 7 kinases), including marked stimulation of cyclin-dependent kinases CDK1 and CDK2 at both 5 and 60 minutes. The other kinases (Fig 3C, top 7 kinases) were downregulated in predicted kinase activity in at least one time point, with AURKB the most repressed. Indeed, AURKB showed the greatest alteration in predicted kinase activity at 60 min of any kinase analyzed. While the phosphorylation and abundance of AURKB were not directly altered by coculture, our pathway analysis identified AURKB as a key Steiner node whose network neighborhood expanded from 5 to 60 minutes after cell mixing (Fig 3D). Since AURKB is the major regulator of mitosis [32] and we had several lines of evidence showing that coculture-induced signaling alters the function of multiple cell cycle regulators, we next endeavored to determine if modulating these kinases affected HIV-1 spread.

### Identifying cellular kinases and phosphatases regulating HIV-1 cell-cell spread

We next sought to determine if any of the above cellular kinases and phosphatases altered by cell contact affected HIV-1 spread. Since many of these enzymes are essential, gene deletion would not be possible, and blocking expression by siRNA, CRISPR/Cas9, etc. requires significant time for protein depletion, expanding secondary, off-target effects. Accordingly, we focused on specific chemical inhibitors that block kinase or phosphatase activity rapidly, consistent with the relatively short time frame of VS signaling. Of the enzymes identified in Table 2 and Fig 3 with known roles in the cell cycle or transcription, 12 (AURKB, CDK1, CDK9, DYRK1A, EEF2K, LCK, ULK1, WEE1, WNK1, DUSP3, PTPN1, PTPRC) had accessible, selective, small molecule inhibitors (summarized in Table 1). We included a ROCK kinase inhibitor as a positive control, as inhibiting ROCK is known to inhibit HIV-1 cell to cell spread [93]. Co-culture-induced effects included changes in the phosphorylation of Ser 2 of the RNA pol II C-terminal domain, a target of both CDK9 (a known effector of HIV-1 transcription [92,94]) and CDK13 [95]. To test for possible significant effects of this modification, both the above-noted CDK9 inhibitor and a CDK13 inhibitor were included.

**Fig 4:**
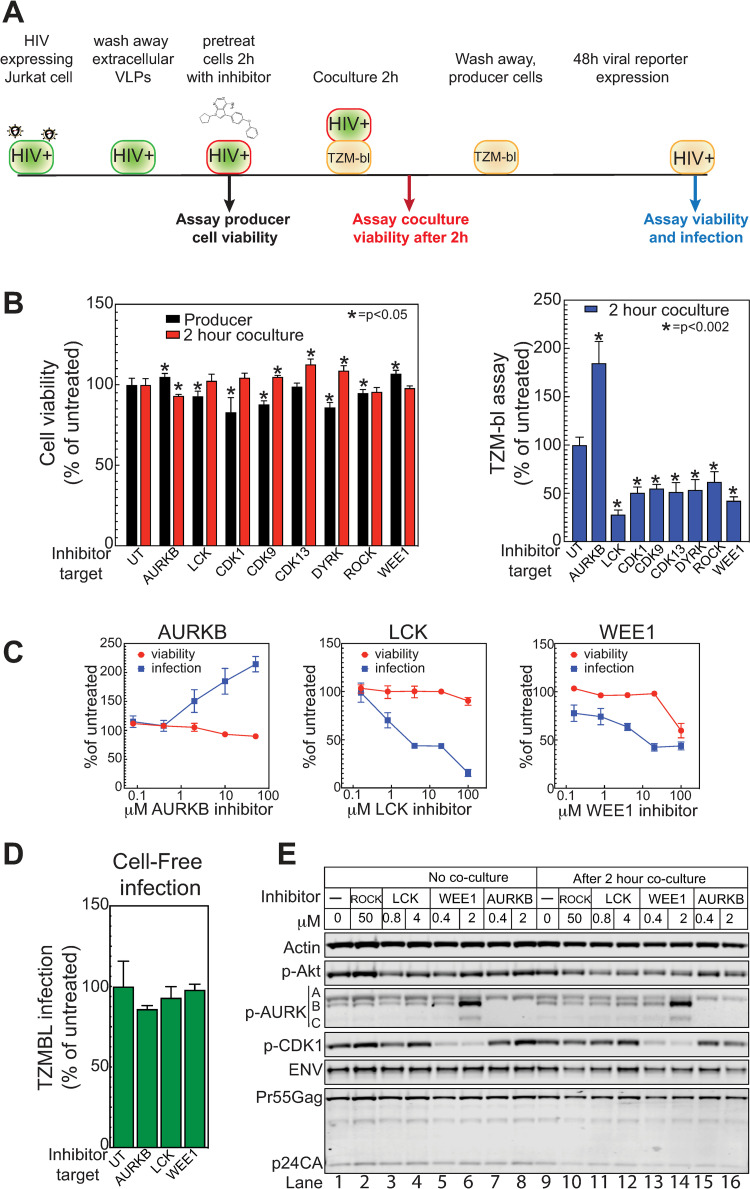
Inhibition of cellular kinases alters cell to cell mediated viral spread. (A) Schematic of TZM-bl assay. HIV expressing producer cells are pelleted and washed to remove extracellular viral like particles (VLP). The cells were resuspended in media containing kinase inhibitor and incubated for 2hr. A sample was removed and tested for effects of the chemical on producer cell viability. Treated cells were then mixed with TZM-bl reporter cells (HeLa cells that express the HIV CD4 receptor and co-receptors and have a TAT responsive promoter upstream of the firefly luciferase reporter), diluting the chemical 5-fold and incubated for 2 hours. The treated producer cells were then removed from the TZM-bl cells by washing and the cells were incubated for 48 hours to allow for infection and reporter gene expression. At 48 hours, cell viability and viral transfer were assayed. (B) The effects of kinase inhibitors targeting AURKB (Barasertib, 10 μM), LCK (LCK inhibitor, 100 μM), CDK1 (RO-3306, 20 μM) CDK9 (LDC000067, 10 μM), CDK13 (THZ531, 2 μM), DYRK1A (Harmine, 50 μM), ROCK1 (Y-27632, 50 μM) and WEE1 (MK1775, 20 μM) relative to untreated (UT) on cell viability (left panel) and TZM-bl cell activation (right panel) were assayed via a mitochondrial ATP assay (Cell titer-glo, Promega) and firefly luciferase (Bright-glo, Promega), respectively. (C) Dose response effects of inhibitors to selected kinases to cell-to-cell HIV spread and cell viability. (D) HIV expressing producer cells were pelleted and washed to remove extracellular VLPs. The cells were resuspended in media containing kinase inhibitor as described in (B) and incubated for 2hr. Treated cells were then pelleted and the VLP containing supernatants mixed with TZM-bl reporter cells, diluting the chemical 5-fold, and incubated for 48 hrs to allow for infection and reporter gene expression. (E) Western blot of HIV producer cells after 2-hour treatment with indicated inhibitors. Cells were mixed with target cells for two hours, then washed off the target cells, collected and processed for western blotting with the indicated antibodies. (A-D) The data shown are the average mean values obtained in an experiment performed with quadruplicate samples and are representative of three independent experiments. Error bars indicate the standard deviation of the data in all panels. P-values were calculated using a standard Student’s t-test and significant changes relative to untreated are indicated.

We next tested the effects of inhibiting selected kinases on Env / CD4-driven cell-cell interaction, using a series of assays starting with interaction of HIV-1+ Jurkat cells with the TZM-bl reporter cell line (S6 Fig). TZM-bl cells are HeLa cells that express high levels of CD4 and HIV-1 coreceptors CXCR4 and CCR5, and carry an integrated reporter cassette expressing the firefly luciferase gene under the control of the HIV-1 LTR promoter. This LTR promoter is activated by the viral TAT transcription factor, which must be transferred from the HIV-1-producing Jurkat cells to TZM-bl cells via infection or membrane fusion-mediated cell content mixing, as dissected in more detail below. As an initial assay, Jurkat cells generating infectious HIV-1 were pretreated with each selected kinase inhibitor for 2 hours prior to removing an aliquot for viability analysis, washed to remove extracellular virions, and then cocultured for 2 hours with CD4+ TZM-bl reporter cells (Fig 4A). After 2-hour co-culture, the non-adherent Jurkat cells were removed by washing and the adherent TZM-bl target cells incubated to allow any virions transferred to establish infection. At 48 hours, the TZM-bl cells were assayed for viability and for coculture-induced expression of firefly luciferase. Several inhibitors were rejected due to effects on the viability of the Jurkat producer cells or TZM-bl cells, or had no effect in the spread assay (Table 1 and Fig 4B). However, the inhibitors of AURKB (barasertib), LCK, CDK1, CDK9, CDK13, DYRK, ROCK and WEE1 all modulated co-culture-induced TZM-bl luciferase expression (Fig 4B, right panel) without affecting producer or TZM-bl cell viability (Fig 4B, left panel). Most of the inhibitors decreased co-culture-induced TZM-bl response 2- to 3-fold, while inhibiting AURKB using barasertib enhanced TZM-bl response 2-fold.

We further analyzed AURKB and WEE1 as representative members of the implicated cell cycle regulatory kinases [32,96] with opposite effects on HIV-1 spread. WEE1 has also been reported to be activated by HIV-1 Vpr [33]. We also included LCK, which was reported to affect cell-to-cell spread in target cells [34] and is critical for T cell signaling. Inhibitor dose response experiments in the TZM-bl assay confirmed and extended our initial results (Fig 4C). As before, AURKB and LCK inhibitors showed little to no toxicity, while the Wee1 inhibitor only showed toxicity at the highest concentration. Inhibiting AURKB yielded dose-dependent increases in viral spread, while inhibiting LCK or Wee1 reduced spread.

To see if these kinase-dependent responses might reflect infection by cell-free HIV-1 virions, rather than TZM-bl interaction with the co-cultured HIV-1+ Jurkat cells, we tested the effect of these inhibitors on Jurkat cell production of released HIV-1 virions and their ability to infect TZM-bl cells (Fig 4D). Importantly, all three inhibitors had no significant effect on cell-free infection (Fig 4D), indicating the target kinases exerted their effects through cell-to-cell interaction.

We also confirmed that the inhibitors blocked their expected targets in HIV-1 producer cells. HIV-expressing Jurkat cells were pre-incubated for 2 hours with inhibitor, then co-cultured for 2 hours with TZM-Bl cells. Producer cells were removed, collected by centrifugation, lysed and analyzed by western blotting for relevant kinase targets (AKT for LCK, CDK1 for Wee1 and autophosphorylation for AURKB). Co-culture alone had little effect on the protein signals (Fig 4E, lanes 1 and 9), suggesting that changes induced by cell mixing did not affect total protein levels, were not detected by the antibodies chosen (e.g. a different phosphorylation site), or were due to other alterations, such as subcellular localization (see below). Consistent with the MS results (see above), levels of viral proteins Gag-pr55, Gag-p24CA and Env were unaltered, so that changes in rates of viral spread were not due to gain or loss of viral proteins. LCK inhibition had a modest effect on p-AKT and CDK1 (Fig 4E, lanes 3, 4 and 11, 12). Inhibition of WEE1 yielded an expected reduction in p-CDK1 (Fig 4E, lanes 5, 6 and 13, 14) and, unexpectedly, a significant increase in p-AURKB and p-AURKC levels (Fig 4E, lanes 6 and 14). Accordingly, the effects of WEE1 inhibition on HIV-1 spread may be mediated through multiple synergistic pathways considering that WEE1 and AURKB inhibition had opposing effects in the TZM-bl assay (Fig 4C). Further, the specificity of barasertib for AURKB activity was confirmed as both 0.4 and 2 mM treatment inhibited the autophosphorylation of AURKB but had little effect on AURKA autophosphorylation (Fig 4E, lane 7,8,15 and 16).

### AURKB but not AURKA inhibition affects HIV-1+ Jurkat / TZM-bl cell interaction

**Fig 5:**
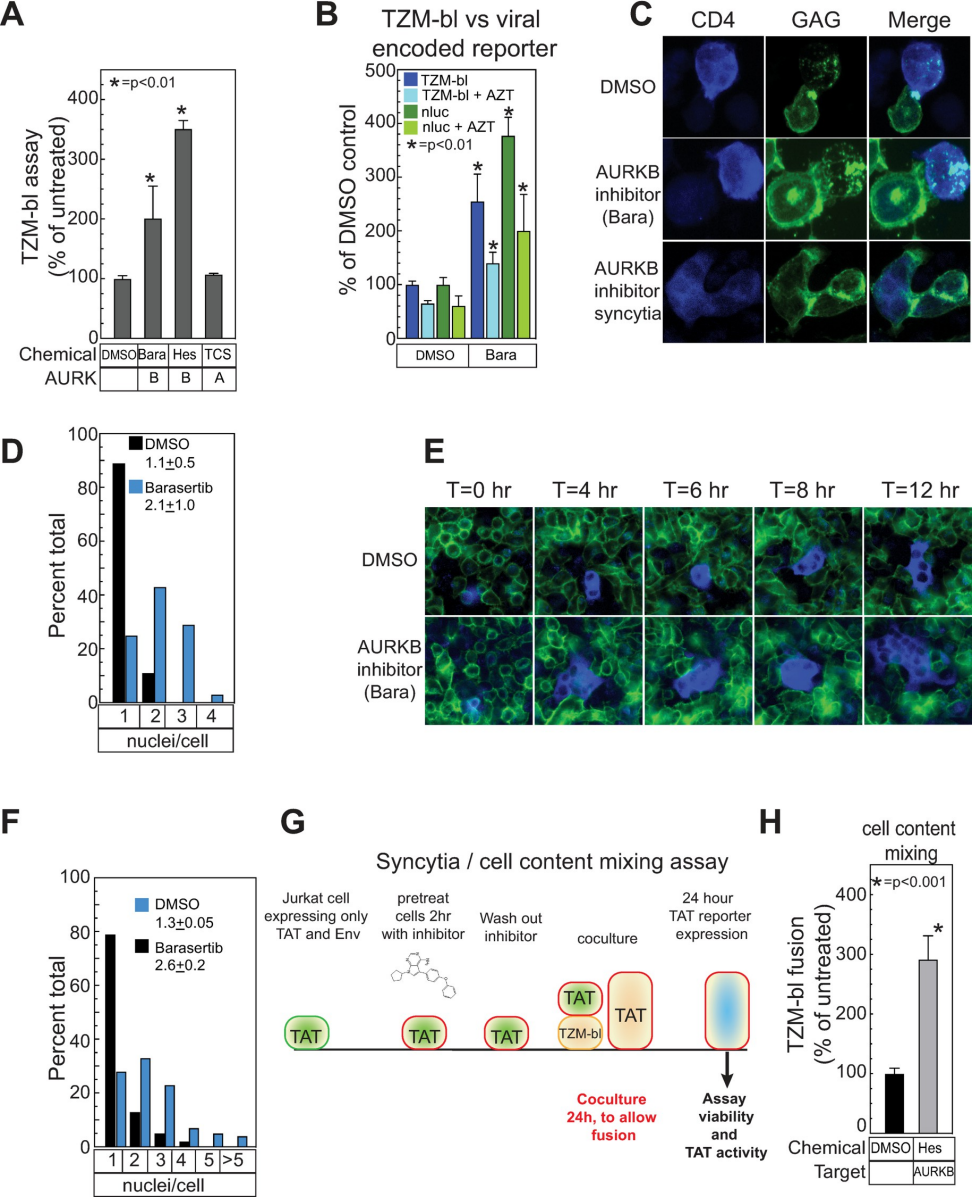
AURKB specifically regulates the fusion activity of the HIV envelope through the cytoplasmic tail domain of Env. (A) The effects of kinase inhibitors targeting AURKB (Barasertib, 20 μM, and Hesperidin, 5 μM) and AURKA (TC-S 7010, 10 μM) was determined as described in Fig 4. (B) HIV-1 Jurkat producer cells treated with DMSO only, 20 μM barasertib and/or 500 nM AZT as indicated were mixed with TZM-bl cells as described in Fig 4 and then assayed for the TZM-bl-encoded firefly luciferase or the virally encoded GFP-NanoLuc (Promega) reporter gene. Barasertib treatment was conducted as described in Fig 4 and the associated figure legend. AZT was added with barasertib and maintained at 500 nM throughout the experiment. (C) HIV producer cells (Jurkat) with a fluorescent protein (CFP) fused to HIV-gag and WT HIV-1 Env were treated for 2h with DMSO or barasertib (20 μM) and mixed with target cells (SupT1) expressing fluorescent CD4 (CD4-YFP) for 20 minutes, fixed, mounted and imaged by confocal microscopy with a 60X objective. Inhibition of AURKB resulted in elongated cell-cell contacts and the frequent formation of syncytia. (D) The distribution of nuclei per cell after treatment with the indicated inhibitors and cell mixing was quantified and normalized as a percentage by counting a minimum of 120 cells from 3 fields from independent experiments are plotted. Mean and SEM are indicated. (E) Selected time frames from live cell images of AURKB inhibitor (Barasertib, 20 μM, 2 hr) -treated Jurkat cells transfected with HIV with a fluorescent protein (CFP) fused to HIV-gag and WT HIV-1 Env seeded on a monolayer of HeLa cells expressing CD4-YFP. (F) The distribution of nuclei per cell 12 hr after treatment and mixing was quantified and normalized as a percentage by counting a minimum of 120 cells from 3 fields from independent experiments. Mean and SEM are indicated in red. (G) Schematic of TZM-bl based syncytia/content mixing assay. Jurkat cells were co-transfected with a plasmid expressing the HIV-1 envelope protein and a second plasmid encoding the HIV transactivator TAT. Since no genome or other viral replication proteins are present, no virions can be formed. The cells were resuspended in media containing kinase inhibitor and incubated for 2hr. Treated cells were then mixed with TZM-bl reporter cells (HeLa cells that express the HIV CD4 receptor and co-receptors and have a TAT responsive promoter upstream of the firefly luciferase reporter). At 24 hours, cell viability and the activity of the TAT-responsive promoter were assayed. Since no virions were produced, the only activation of the TAT-responsive promoter can occur with cell-to-cell fusion and contact mixing. (H) Jurkat cells expressing TAT and WT NL43 Env were treated with an AURKB inhibitor (Hesperidin, 5 μM) as described in (G) and membrane fusion with TZM-bl cells was measured by TAT reporter gene activity. The data shown are the average mean values obtained in an experiment performed with quadruplicate samples and are representative of three independent experiments. Error bars indicate the standard deviation of the data in all panels. P-values were calculated using a standard Student’s t-test and significant changes relative to DMSO treated controls are indicated.

Because the role of LCK in HIV-1 spread was previously characterized [34] and WEE1 inhibition was associated with enhancing AURKB activity (Fig 4E, lanes 6 and 14), we focused on understanding how AURKB limits HIV-1 cell-to-cell interaction. To confirm specificity, we first tested the effects of a second AURKB inhibitor (hesperidin) compared to, as a negative control, a well-studied AURKA inhibitor, TC-S 7010 (Fig 5A). Consistent with our earlier results (Fig 4B and 4C), AURKB inhibition with Hersperidin enhanced co-culture-induced TZM-bl response while TC-S 7010 had no effect (Fig 5A), confirming that inhibiting AURKB activity modulates the TZM-bl assay specifically.

### AURKB modulates HIV-1 envelope fusogenicity at cell-cell contacts

Fig 4D above showed that the effects of AURKB, WEE1 and LCK inhibitors on coculture-based TZM-bl assays were independent of cell-free virion infection, and must represent effects on or downstream of CD4+ TZM-bl interaction with HIV-1-producing Jurkat cells. In principle, TAT might be transferred through such interactions by direct cell-to-cell virion transfer to infect TZM-bl cells and express TAT, or by transferring pre-synthesized TAT from Jurkat to TZM-bl cells by Env-mediated fusion of whole cells or local cell-cell contacts, allowing cytosolic mixing. All of these mechanisms occur in association with VS formation [37], and all could report changes in cell membrane interaction, fusogenicity or other factors affecting VS formation and cell-to-cell spread.

To explore the potential contributions of these mechanisms we extended the basic TZM-bl assay results with complementary assays (S6 Fig), including treatment with the reverse transcriptase inhibitor azidothymidine (AZT) (Fig 5B). In controls lacking a kinase inhibitor, AZT treatment inhibited the TZM-bl assay response by 33–40%, showing that 33–40% of the signal was due to reverse transcription-dependent HIV-1 infection and 60–67% was due to cytosolic mixing through plasma membrane fusion. When Jurkat producer cells were treated with AURKB inhibitor barasertib as in Fig 4A, the TZM-bl signal was stimulated two-fold or more, as before, and 50% of this signal was AZT-sensitive (Fig 5B). Thus, under these co-culture conditions both infection transmission and cytosolic mixing were significant, and the extent of both was stimulated by inhibiting AURKB. Assaying an HIV-1-expressed GFP-NanoLuc reporter in the target cells after washing away the producer cells (following the mixing procedure in Fig 4A) closely paralleled the TZM-bl-encoded firefly luciferase reporter findings (Fig 5B).

Motivated by these findings, we examined the effects of AURKB inhibition on cell-cell interactions by confocal microscopy. Jurkat cells expressing HIV-1 Env and an HIV-1 genome encoding a MA-CFP fusion protein were incubated 2 hours with AURKB inhibitor, mixed 20 minutes on poly-D-lysine coated coverslips with target SupT1 cells expressing a CD4-YFP fusion protein, fixed, mounted and imaged. Control DMSO only-treated cells showed tight VS formation with close overlap of the CD4-YFP and MA-CFP fusion proteins at the cell junctions (Fig 5C, top), confirming VS formation under the conditions of the MS proteomics experiments. Treatment with AURKB inhibitor barasertib expanded the area of cell-to-cell contacts and potential membrane exchange (Fig 5C, middle), often resulting in membrane fusion and formation of multinucleated syncytia (Fig 5C, bottom). Barasertib treatment increased the average number of nuclei per cell in the mixed T cell population from 1.1 ± 0.5 to 2.1 ± 1.0 (Fig 5D). These data indicate that AURKB may modulate membrane dynamics or HIV-1 Env fusion activity and, together with the AZT results of Fig 5B, show that inhibiting AURKB increases activity in the TZM-bl assay as much by inducing syncytia as by increasing infection spread between distinct cells.

To confirm syncytia formation, we performed time course, live-cell, video microscopy by mixing Jurkat cells transfected with an HIV-1 genome expressing MA-CFP fusion protein and WT Env with HeLa cells stably expressing CD4-YFP. Strikingly, inhibiting AURKB resulted in the rapid accumulation and spread of large, heavily multinucleated syncytia from single initially infected cells, far in excess of rarer, smaller syncytia that formed in DMSO treatment (Fig 5E). Quantitation revealed that the average number of nuclei per infected cell increased from 1.3 ± 0.5 to 2.6 ± 0.2 after barasertib treatment (Fig 5F). Although 90% of the cells exhibited 1 to 4 nuclei per cell, syncytia as large as 15 nuclei were observed (Fig 5F).

To further measure the increase in membrane fusion activity, we modified the viral spread assay (Fig 4A) into a cell content mixing assay (Fig 5G) by transfecting Jurkat cells not with a full HIV-1 genome but only with plasmids expressing TAT and HIV-1 Env. Since no HIV-1 genome plasmid was included, Jurkat-expressed TAT could only be delivered to a TZM-bl cell if the two cell types fused. Cells expressing TAT and Env were pre-treated with DMSO or AURKB inhibitor, washed, and co-cultured 48 hours with TZM-bl target cells to allow content mixing and luciferase expression. Treating TAT- and Env-expressing cells with AURKB inhibitor enhanced content mixing 3-fold (Fig 5H). Thus, AURKB’s effect requires only the HIV-1 envelope protein and enhances Env fusion activity.

### HIV-1 Env cytoplasmic tail domain is required for AURKB responsiveness

**Fig 6:**
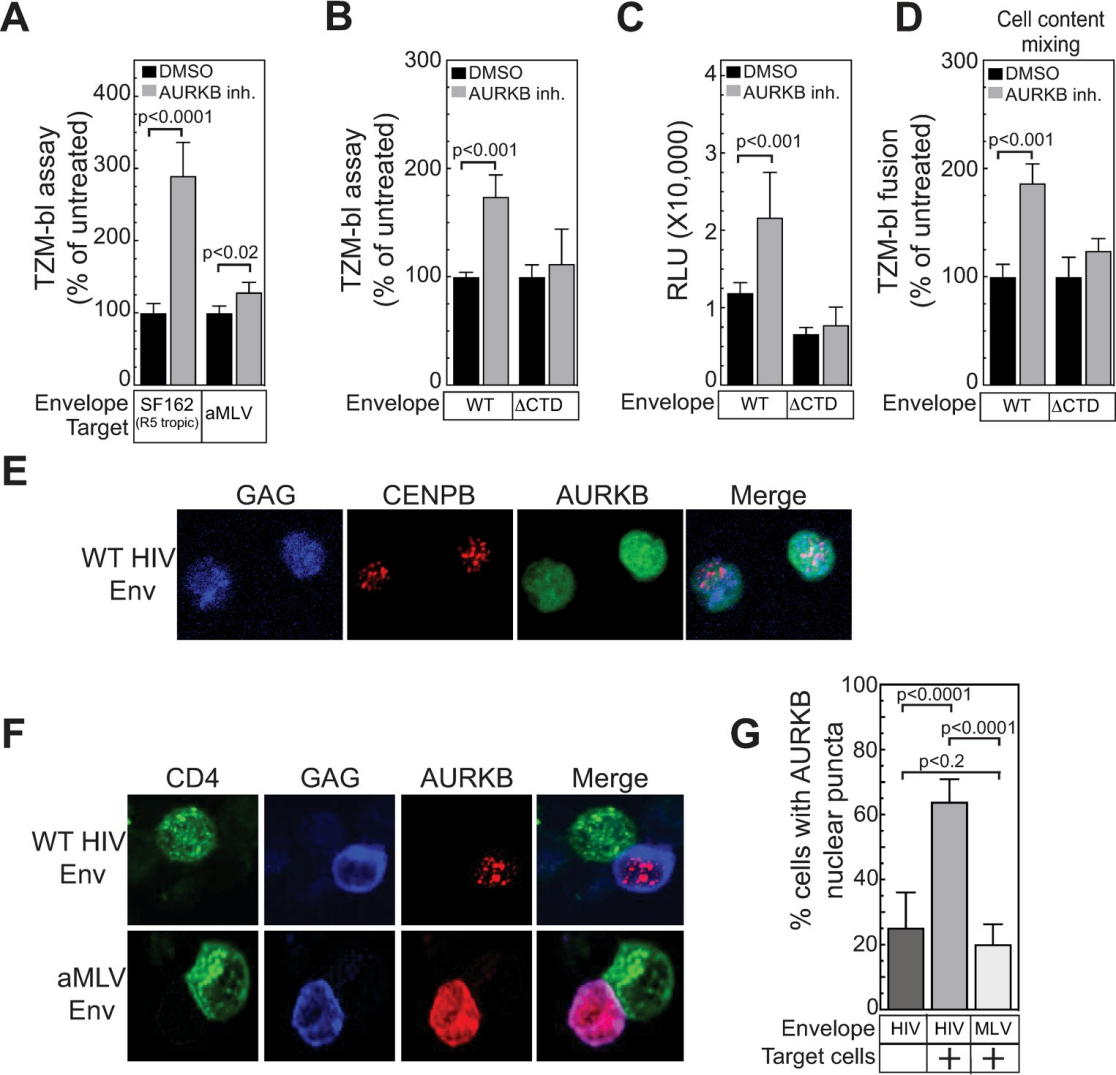
Interaction of WT HIV Env-expressing cells with CD4+ cells relocalizes AURKB to nuclear puncta. (A) The effect of AURKB inhibition (Hesperidin, 5 μM) on viral spread was determined as described in Fig 4, except the CCR5 tropic envelope from HIV-1 strain SF162 or the envelope from amphotropic MLV was used instead of the envelope from the CXCR4 HIV-1 strain NL4-3. (B) The effect of AURKB inhibition (Barasertib, 20 μM) on viral spread was determined as described in Fig 4 with cells expressing the NL43 genome and either the WT envelope or a variant lacking the cytoplasmic tail domain (ΔCTD) derived from the envelope of CXCR4 HIV-1 strain NL4-3. (C) The firefly luciferase activities from the experiment in (B) are reported as raw, unprocessed relative light units (RLU) instead of normalized to 100%. (D) The effect of AURKB inhibition (Barasertib, 20 μM) on syncytia formation was determined as described in Fig 5G with cells expressing HIV-1 TAT and either the WT envelope or a variant lacking the cytoplasmic tail domain (ΔCTD) derived from the envelope of CXCR4 HIV-1 strain NL4-3. The data shown in (A-D) are the average mean values obtained in an experiment performed with quadruplicate samples and are representative of three independent experiments. (E) HIV producer cells (Jurkat) were transfected with a plasmid encoding mCherry-AURKB and plasmids containing an HIV genome encoding fluorescent protein (CFP) fused to HIV-gag, and a plasmid WT HIV envelope and were allowed to settle to a poly-D-lysine slide for 20 minutes, fixed, mounted and imaged by confocal microscopy with a 60X objective. (F) HIV producer cells (Jurkat) were transfected with a plasmid encoding mCherry-AURKB and plasmids containing an HIV genome encoding fluorescent protein (CFP) fused to HIV-gag, and plasmids encoding either amphotropic MLV or WT HIV envelope and mixed with target cells (SupT1) expressing fluorescent CD4 (CD4-YFP) for 20 minutes, fixed, mounted and imaged by confocal microscopy with a 60X objective.(G) Total AURKB localization at nuclear puncta by WT HIV-1 or MLV envelope after mixing with target cells. 10 individual fields were counted and transfected cells with AURKB puncta were counted. The data shown are the average mean values from 10 independent fields and are representative of three independent experiments. Error bars indicate the standard deviation of the data in all panels. P-values were calculated using a standard Student’s t-test and significant changes relative to DMSO treated or no target cell controls are indicated.

The experiments above (Fig 5) were performed with HIV-1 NL4-3 Env, which uses the CXCR4 co-receptor. To test if the AURKB effect was specific to HIV Env or co-receptor usage, we compared the TZM-bl response induced by co-culture with Jurkat cells expressing viruses pseudotyped with CCR5-tropic (SF162) HIV-1 Env protein or envelope from amphotropic murine leukemia virus (aMLV). AURKB inhibition enhanced spread with the SF162 envelope 2.8-fold, but only a not statistically significant 1.2-fold increase was observed with aMLV envelope protein (Fig 6A).

Since the cytoplasmic tail domain (CTD) of HIV-1 env has been implicated in signaling [36], we tested if the CTD affected the response to AURKB inhibition. Deleting the CTD reduced the effect of AURKB inhibition from ~2-fold to a not statistically significant 1.2-fold (Fig 6A–6C). Depending on the particular truncation point, some Env CTD deletions can increase cell-cell fusion by up to 2-fold [97,98], which might partially saturate the TZM-bl assay and thereby reduce responsiveness to AURKB inhibition. However, as shown by the un-normalized luciferase reporter values in Fig 6C, the CTD deletion used here moderately reduces the TZM-bl assay response, removing this concern. Consistent with these findings and our observation that AURKB regulates Env fusion activity (Fig 5), deleting the CTD similarly eliminated responsiveness to AURKB inhibition in the cell content mixing/syncytia assay (Fig 6D). Thus, AURKB specifically affects HIV-1 envelope fusion activity in a CTD-dependent but co-receptor-independent manner.

### HIV-1 Env CTD induces AURKB relocalization to nuclear puncta after Env:CD4 interaction

Since AURKB localization is a major factor in regulating its substrate specificity [32], we determined if Env+:CD4+ cell-cell interaction affected AURKB localization. Prior to co-culture, AURKB in WT NL43-transfected Jurkat producer cells is dispersed throughout the nucleus and cytosol (Fig 6E). To define the effects of co-culture, Jurkat cells co-transfected with plasmids expressing an HIV genome encoding MA-CFP, mCherry-AURKB and either WT NL4-3 HIV-1 Env or aMLV Env were mixed with SupT1 cells expressing CD4-YFP for 20 min on poly-D-lysine coated slides, fixed and imaged by confocal microscopy. Strikingly, in cells expressing HIV-1 Env, but not in cells expressing aMLV Env, AURKB relocalized from its initial dispersed cytoplasmic and nuclear distribution to distinct nuclear puncta (Fig 6F, third column). Quantitation showed that cell populations expressing HIV Env, when mixed with CD4+ target cells, had 2.6-fold more cells with AURKB nuclear puncta than unmixed cells and 3.2-fold more than cells expressing MLV env mixed with CD4+ target cells (Fig 6F and 6G). Thus, nuclear relocalization of AURKB is induced by the interaction between HIV-1 Env on producer cells with CD4 on target cells.

**Fig 7:**
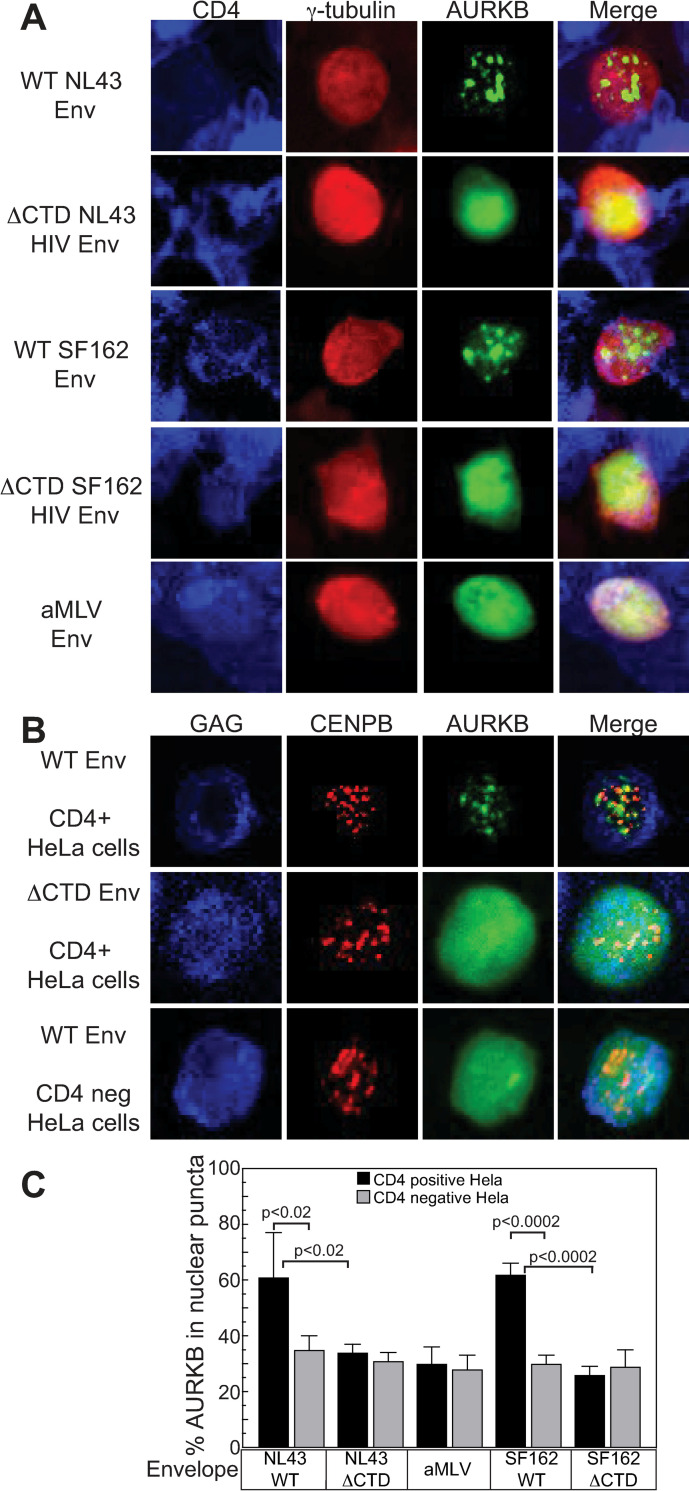
HIV Env CTD relocalizes AURKB adjacent to inner centromeres. (A) Jurkat cells expressing a fluorescent AURKB, fluorescent γ-tubulin and full length or ΔCTD variants of a CXCR4 HIV envelope (NL43), a CCR5 tropic HIV envelope (SF162) or amphotropic MLV envelope were mixed for 20 minutes with HeLa cells expressing fluorescent CD4-CFP, fixed, mounted and imaged at 60X by confocal microscopy. (B) HIV producer cells (Jurkat) were co-transfected with plasmids containing an HIV genome expressing the HIV matrix fused to a fluorescent protein (iRFP670) and plasmids encoding WT env or a variant lacking the cytoplasmic tail domain (CTD) and plasmids encoding a fluorescent (GFP) AURKB fusion and plasmids encoding fluorescently tagged (mCherry) variants of the inner centromere protein CENPB. Transfected cells were mixed with cells expressing the HIV receptor and co-receptors (TZM-bl) or receptor negative (HeLa) cells for 20 minutes, fixed, mounted and imaged at 60X by confocal microscopy. Consistent with relocalization to the CPC, AURKB relocalized to puncta adjacent to CENPB puncta. This relocalization requires the Env CTD and receptor on target cells. (C) Quantitation of AURKB relocalization. HIV producer cells (Jurkat) were co-transfected with plasmids containing an HIV genome and plasmids encoding X4 tropic (NL43) or R5 tropic (SF162) WT env, a variant lacking the cytoplasmic tail domain (CTD), or amphotropic MLV envelope along with plasmids encoding fluorescent fusion proteins GFP-AURKB and mCherry-CENPB. Transfected cells were mixed with cells expressing the HIV receptor and co-receptors (TZM-bl) or receptor free cells (HeLa) cells for 20 minutes, fixed, mounted and imaged at 60X by confocal microscopy. 10 individual fields were counted and transfected cells with AURKB puncta were counted. The data shown are the average mean values from 10 independent fields and are representative of three independent experiments. Error bars indicate the standard deviation of the data in all panels. P-values were calculated using a standard Student’s t-test and significant changes between CD4 expressing HeLa cells relative to CD4 negative HeLa are indicated.

To follow up on our results that AURKB effects on Env fusion were independent of co-receptor type and mediated by the CTD (Fig 6A–6D), envelope expression plasmids for either WT or ΔCTD variants of the CXCR4 NL4-3 or CCR5 SF162 HIV-1 Env or WT aMLV Env were each co-transfected into Jurkat cells together with plasmids expressing the HIV genome, GFP-AURKB and mCherry-γ-tubulin- to mark transfected cells. Transfected cells were added to a monolayer of HeLa cells expressing CD4-CFP for 20 min, fixed and imaged by confocal microscopy. Consistent with our finding above that the HIV-1 Env CTD is required to mediate AURKB-linked effects on HIV-1 spread (Fig 6B–6D), AURKB relocalized to discrete nuclear puncta with WT NL4-3 and SF162 HIV-1 Env but not with the ΔCTD variants or aMLV Env (Fig 7A). Thus, the CTD of HIV-1 envelope mediates AURKB relocalization to nuclear puncta, in correlation with AURKB effects on HIV-1 infection spread.

### AURKB relocalizes to centromeres after coculture of HIV-1+ and CD4+ cells

During mitosis, AURKB associates with the chromosomal passenger complex (CPC) and is targeted to centromeres [32]. Accordingly, we examined if Env-CD4 interaction-induced AURKB localization to nuclear puncta also involved the centrosome. Jurkat cells were co-transfected with three plasmids expressing (i) an HIV genome encoding MA-CFP and either WT or ΔCTD NL4-3 Env, (ii) GFP-AURKB and (iii) an mCherry fusion to inner centromere protein CENPB (Fig 7B). To measure possible variations from different cell cycle stages, etc., in these unsynchronized populations, we scored AURKB localization under each condition in a minimum of 100 cells across 5 randomly selected fields. When Jurkat cells expressing WT Env were mixed with CD4-expressing target cells, 60% of Jurkat cells showed AURKB re-localization from its typically diffuse distribution to nuclear puncta, primarily adjacent to or colocalized with the inner centromere CENPB protein, consistent with AURKB localization when part of the CPC [99] (Fig 7B, top row and Fig 7C). In contrast, upon expression of ΔCTD Env or in the absence of CD4 on target cells, AURKB remained diffuse in 70% of Jurkat cells (Fig 7B, middle and bottom rows, and Fig 7C). Thus, Env-CD4 interaction dramatically promotes AURKB relocalization to centromeres in cell populations dispersed across the cell cycle.

**Fig 8:**
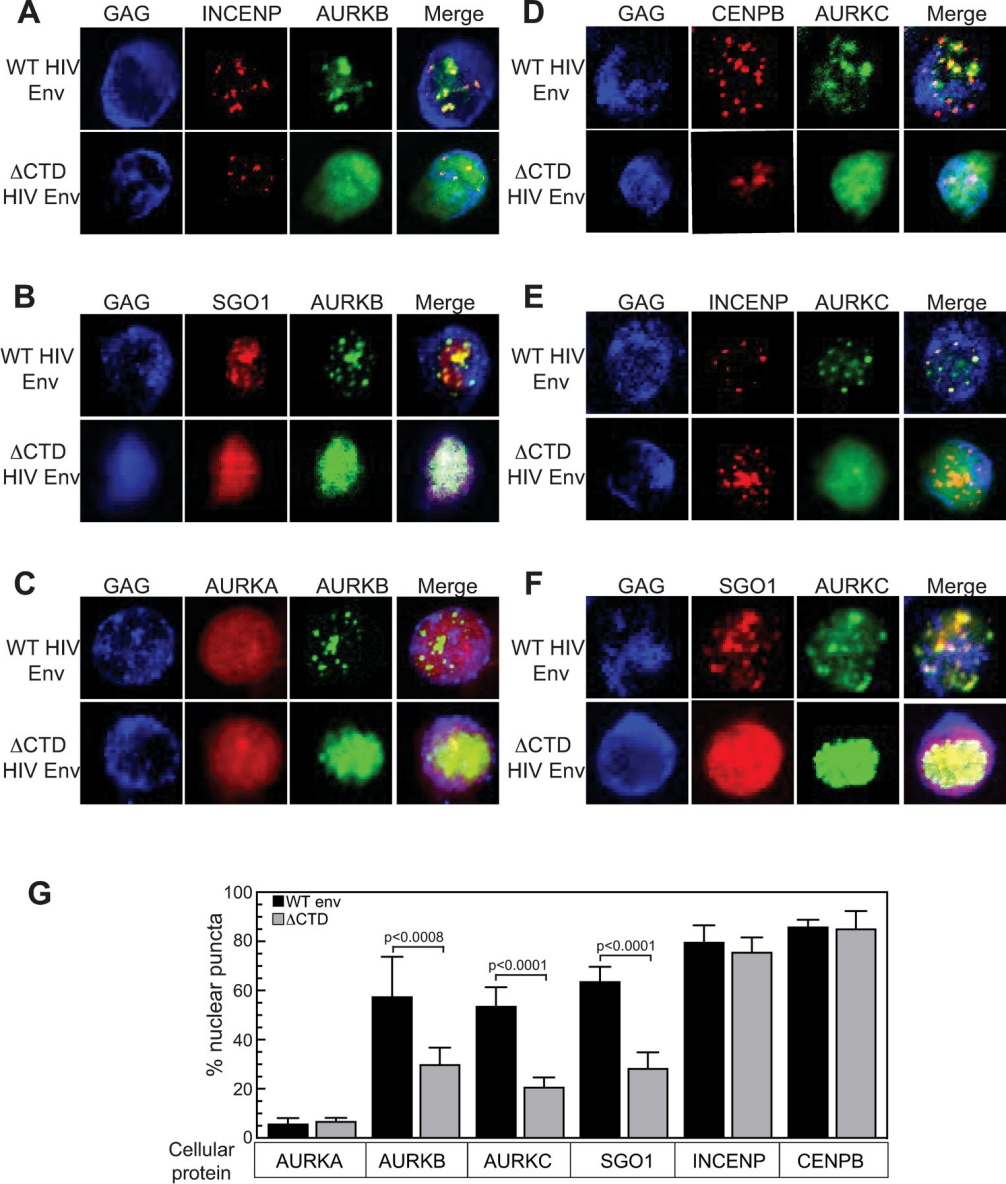
HIV Env CTD relocalizes AURKB and SGO1 to the chromosomal passenger complex (CPC) adjacent to inner centromeres. HIV producer cells (Jurkat) were co-transfected with plasmids containing an HIV genome expressing the HIV gag fused to a fluorescent protein (iRFP670) and plasmids expressing WT env or a variant lacking the cytoplasmic tail domain (CTD) along with plasmids expressing the indicated fluorescent fusion proteins (A) GFP-AURKB and mCherry-INCENP fusion and (B) GFP-AURKB and mCherry-SGO1 or (C) GFP-AURKB and mCherry-AURKA. (D) GFP-AURKC and mCherry-CENPB, (E) GFP-AURKC and mCherry-INCENP, (F) GFP-AURKC and mCherry-SGO1 fusion. Transfected cells were mixed with cells expressing the HIV receptor CD4 and its co-receptors (TZM-bl cells) for 20 minutes, fixed, mounted and imaged at 60X by confocal microscopy. (G) Percentage of cells transfected with the indicated florescent protein showing that protein localized in nuclear puncta. The data shown are the average mean values from 10 independent fields and are representative of three independent experiments. Error bars indicate the standard deviation of the data in all panels. P-values were calculated using a standard Student’s t-test and significant changes between CD4 expressing HeLa cells relative to CD4 negative HeLa are indicated. Consistent with relocalization to the CPC, AURKB, AURKC and SGO1 relocalized to puncta adjacent to CENPB puncta. This relocalization requires the Env CTD and receptor on target cells.

To further confirm that Env-CD4 signaling targets AURKB to the centromere, we examined the localization of another CPC subunit and known AURKB interaction partner, INCENP (Fig 8A), and the CPC regulatory protein SGO1 (Fig 8B). When Jurkat cells expressing WT Env were mixed with CD4-expressing target cells, GFP-AURKB colocalized to mCherry-INCENP-containing nuclear puncta in cells expressing WT Env but not a ΔCTD Env (Fig 8A). Interestingly, both CENPB (Fig 7B) and INCENP (Fig 8A) were at nuclear puncta regardless of the Env variant expressed. In contrast, both mCherry-SGO1 and GFP-AURKB relocalized to substantially co-localized nuclear puncta in the presence of WT Env but not with ΔCTD Env (Fig 8B), suggesting that SGO1 may undergo similar regulation as AURKB or that AURKB may bring SGO1 to the CPC. Taken together, interaction of Env+ and CD4+ cells induces relocalization of AURKB to the CPC at centromere.

Three Aurora kinases family members are encoded in the mammalian genome. AURKA interacts with TPX2 and regulates centrosome function, mitotic entry and spindle assembly. AURKB is part of the CPC and participates in chromatin modification, microtubule attachment to the kinetochore, spindle checkpoint and cytokinesis. AURKC is closely related to AURKB but is primarily expressed in germ cells [32], as well as to low but detectable levels in SupT1 and Jurkat cell lines (Fig 4E). To determine if Env-CD4 interaction relocalized other Aurora kinase family members, we transfected Jurkat cells with plasmids expressing an HIV genome encoding a fluorescent GAG-CFP, WT or ΔCTD Env, GFP-AURKB and a plasmid encoding mCherry-AURKA (Fig 8C). Transfected cells were mixed with CD4-expressing HeLa cells 24 hours post transfection, fixed and imaged by confocal microscopy. While AURKB relocalized to nuclear puncta in WT Env expressing cells but remained diffuse in ΔCTD-Env expressing cells, AURKA remained diffuse under either condition (Fig 8C). Thus, AURKA localization is unaffected by Env-CD4 interaction. In contrast, GFP-AURKC relocalized to mCherry-CENPB- (Fig 8D) and mCherry-INCENP- (Fig 8E) containing nuclear puncta in in the presence of WT Env but remained diffuse in ΔCTD Env-expressing cells. Similarly, both AURKC and SGO1 relocalized to overlapping nuclear puncta in a CTD-dependent manner (Fig 8F). Thus, SGO1, AURKB and the closely related AURKC, but not the more distantly related AURKA exhibited a 2-fold or greater increase in localization to nuclear puncta mediated by CD4 induced signaling through the HIV-1 Env CTD (Fig 8G).

### HIV-1 Env:CD4 interaction is necessary and sufficient to induce premature AURKB centromeric localization

**Fig 9:**
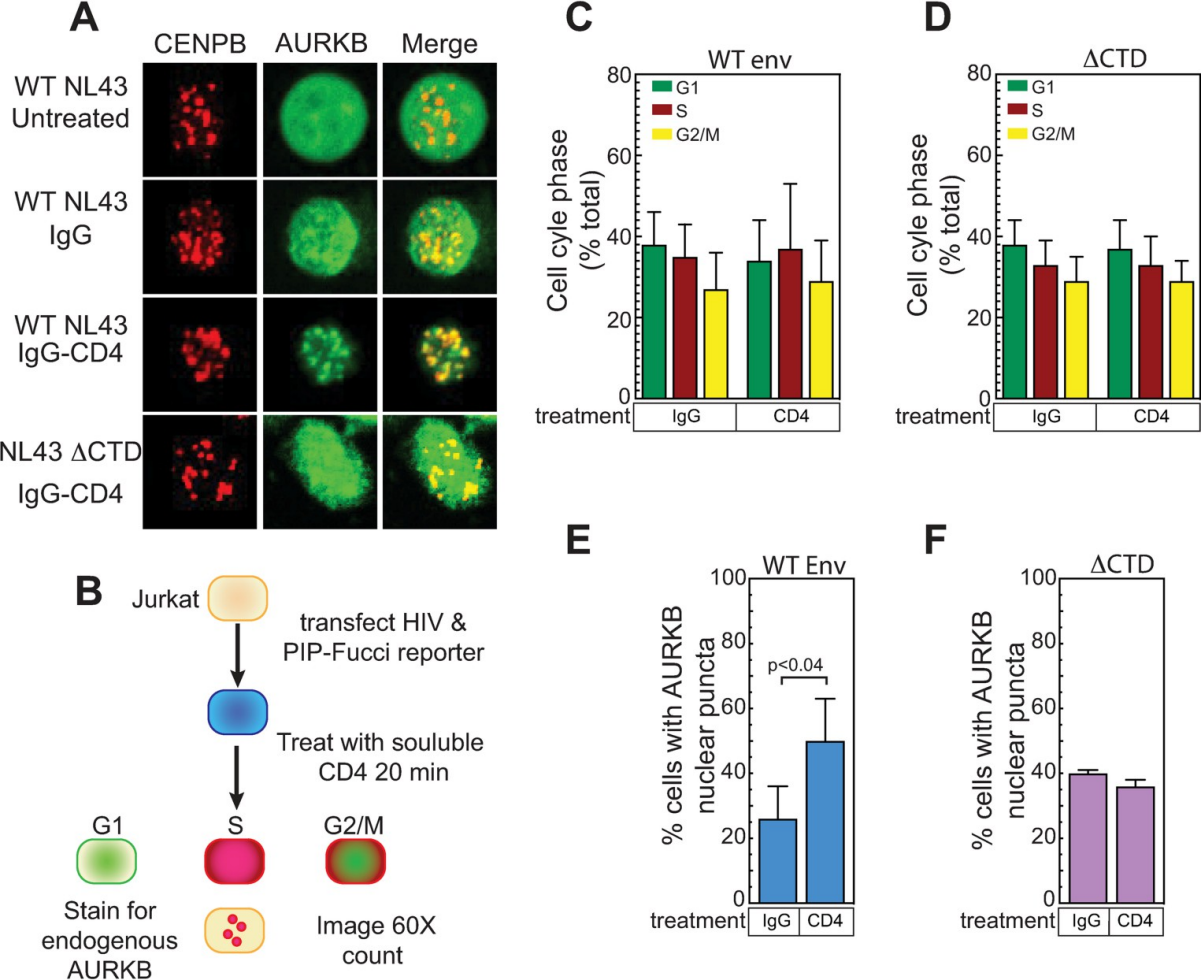
Interaction with soluble CD4 HIV Env is sufficient to relocalize AURKB. (A) Jurkat cells were co-transfected with plasmids encoding the indicated HIV envelope, GFP-AURKB and mCherry-CENBP. 24 hours post transfection, cells were incubated with purified IgG or soluble IgG-CD4 fusion protein for 20 minutes, fixed, mounted and imaged at 60X by confocal microscopy. Treatment with the soluble CD4 fusion protein caused HIV to relocalized to CENBP adjacent foci. (B) Schematic for quantitation of CD4 induced AURKB localization and cell cycle changes. Jurkat cells were co-transfected with plasmids encoding the indicated HIV envelope, along with mAzurite-Histone H2B to mark the transfected cells and a plasmid that encodes the 2-color PIP-Fucci system than uses a YFP-PIP protein to mark G1 cells, an mCherry-Geminin to mark S-phase cells. G2/M phase cells are dual positive. 24 hours post transfection, cells were incubated with purified IgG or soluble IgG-CD4 fusion protein for 20 minutes, fixed, stained for endogenous AURKB with a far-red secondary (alexafluor 647), mounted and imaged at 60X by confocal microscopy. 10 independent fields were counted, and transfected cells were scored for CD4 induced changes to the cell cycle stage with (C) WT or (D) ΔCTD envelope. (E) Total AURKB localization at nuclear puncta by WT or (F) ΔCTD envelope. The data shown are the average mean values obtained from three independent experiments with a minimum of 550 cells counted per condition between experiments. Error bars indicate the standard deviation of the data in all panels. P-values were calculated using a standard Student’s t-test and significant changes of IgG treated control cells to soluble CD4 treated cells are indicated.

We next sought to determine if the Env:CD4 interaction was the minimal requirement for AURKB relocalization. Jurkat cells were co-transfected with mCherry-CENPB and WT NL4-3 Env expression plasmids. 24 hours post transfection, cells were placed on a poly-D-lysine coated cover slip and incubated with either human IgG (hIgG) or a soluble CD4-hIgG fusion protein for 20 min. Cells were fixed, mounted and imaged by confocal microscopy. WT HIV-1 Env and soluble CD4-IgG were sufficient to induce AURKB relocalization to centrosomes (Fig 9A).

**Fig 10:**
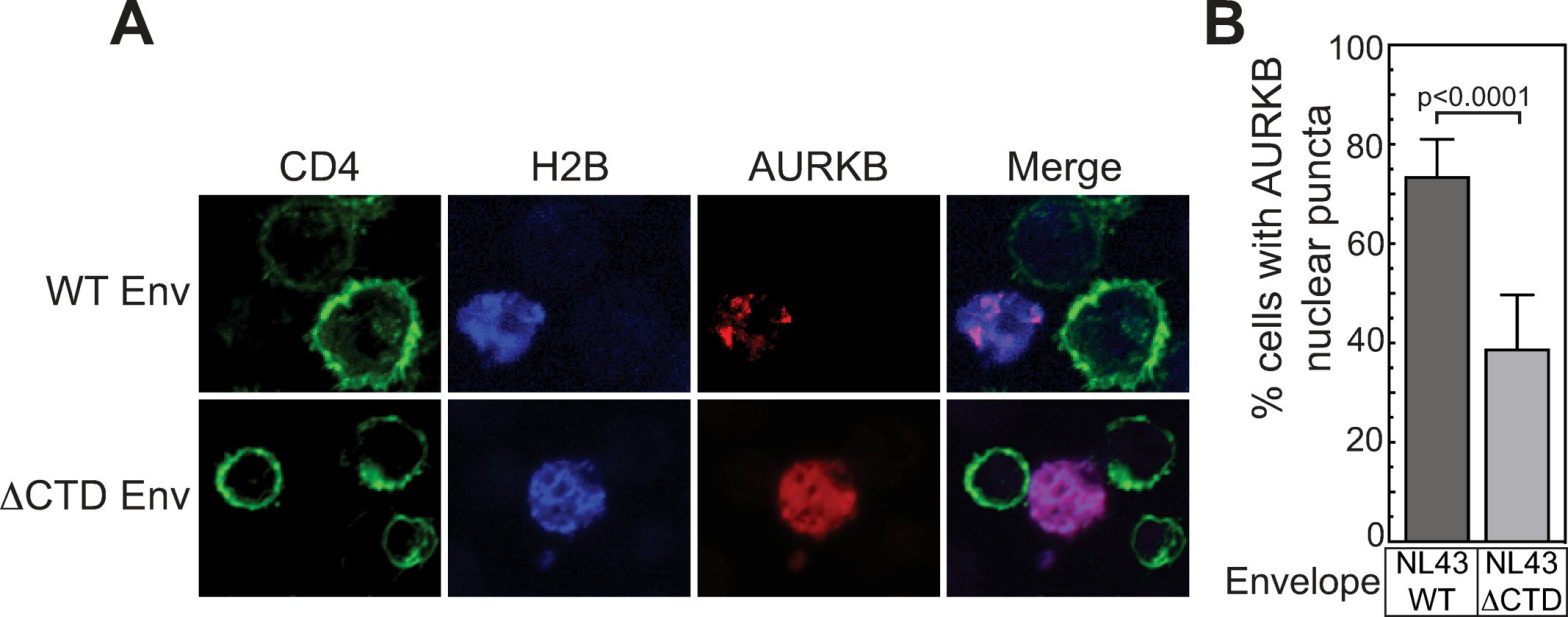
Interaction with CD4+ T cells relocalizes AURKB to nuclear puncta in primary T cells expressing WT but not ΔCTD HIV Env. (A) Primary human T cells were transfected with plasmids expressing mCherry-AURKB, an HIV genome, Blue fluorescent histone H2B, and either WT or ΔCTD HIV envelope. These cells then were mixed with target cells (SupT1) expressing fluorescent CD4 (CD4-YFP) for 20 minutes, fixed, mounted and imaged by confocal microscopy with a 60X objective. (B) Percentage of primary T-cells transfected with mCherry AURKB and WT HIV or ΔCTD HIV envelope as indicated showing AURKB localization at nuclear puncta after mixing with target cells. For each condition the data shown are the average mean values from 10 independent fields and are representative of three independent experiments. Error bars indicate the standard deviation of the data in all panels. P-values were calculated using a standard Student’s t-test and significant changes relative to WT HIV Env expressing cells are indicated.

The ability to induce AURKB relocalization with soluble CD4 facilitated our development of assays to test for possible cell cycle effects of HIV-1 Env-CD4 interaction and their potential correlation with AURKB relocalization (Fig 9B). Jurkat cells were co-transfected with either WT or ΔCTD HIV-1 Env expression plasmids, mAzurite-Histone H2B to mark transfected cells, and the two color PIP-Fucci reporter plasmid [64], which allows delineating cell cycle stages based on YFP-PIP (accumulates in G1, degraded in S) and mCherry-Geminin (accumulates in S, degraded after M). 24 hours post transfection, cells were placed on a poly-D-lysine coated cover slip and incubated with either human IgG (hIgG) or a soluble CD4-hIgG fusion protein for 20 min. Cells were fixed, stained for endogenous AURKB, mounted and imaged by confocal microscopy. For each condition tested, 10 independent imaging fields were collected, and transfected cells were scored for cell cycle stage and AURKB relocalization. At least 150 cells were scored per experiment, and three independent experiments were averaged. Treatment of cells with soluble CD4-IgG did not alter the cell cycle distribution with either WT (Fig 9C) or ΔCTD (Fig 9D) Env-expressing cells. In contrast, soluble CD4-IgG treatment resulted in a significant increase in AURKB relocalization in the WT Env (Fig 9E) but not the ΔCTD (Fig 9F) expressing cells. Finally, we confirmed these results in primary T cells and demonstrated that the HIV Env CTD induces AURKB localization to nuclear puncta (Fig 10). These data indicate that, upon interaction with CD4, WT HIV Env acts through its CTD to simultaneously enhance HIV-1 infection spread and induce centromeric localization of AURKB without affecting the cell cycle. Below the Discussion considers possible mechanisms for these effects.

## Discussion

Here we applied a quantitative proteomic approach combining SILAC and TMT labeling (Fig 1) to define cell-specific changes to protein abundance and phosphosignaling in the context of co-culturing HIV-1-producer and uninfected cell populations. These technical advances for studying mixed cell populations should be broadly applicable to studies of the effects of cell-cell interactions in many normal and pathogenic processes, such as development, cancer and immunological responses. Using these approaches, we explored changes to signaling pathways induced in HIV-1-producer T cells upon contact with uninfected target T cells, to identify host factors that enhance or inhibit infection spread.

We identified altered levels of >20,000 phosphorylated and non-phosphorylated peptides in producer cells with, surprisingly, only minor changes to HIV-1 proteins (Fig 1B) but a remarkable number of major changes to the host cell proteome (S1 and S2 Tables). The number of controls that could be added to the mass spectrometry experiments was severely limited because strong statistical comparisons could only be made within each individual TMT-labeled multiplex of ≤10 samples, which through pooling were all subjected to identical purification and MS treatments, including any loss or enrichment of particular peptides. Nevertheless, our MS controls using an HiV-1 ΔEnv variant and HIV-1 Env only showed that the proteomic and particularly phosphoproteomic changes were largely driven by Env-dependent signaling (Fig 2).

These changes in protein abundance and phosphorylation mainly clustered in three ontological categories; affecting transcription regulatory pathways, immune cell signaling or, unexpectedly, regulation of the cell cycle (Fig 3A and 3B). Because signals are rapidly transmitted from HIV-1 Env:CD4 interactions through phosphorylation cascades [26,34,75], we analyzed coculture-induced changes to kinase or phosphatase activity in producer cells (Fig 3C). We focused on a subset of these pathways, testing in an HIV-1 cell-cell spread assay (Fig 4) the effects of known chemical inhibitors of several cell cycle-relevant kinases (CDK1, DYRK1A, WEE1, and AURKB) compared to known regulators of HIV-1 replication affecting T cell receptor signaling (LCK) or viral transcription (CDK9 and CDK13). HIV-1 cell-to-cell transfer was reduced by specific chemical inhibitors of each of these kinases, with the exception of AURKB, whose inhibition enhanced viral spread.

Of the positive co-factors, DYRK1A is a dual specificity serine/tyrosine kinase involved in the stability of cell cycle regulatory proteins [100,101]. WEE1 is a negative regulator of mitotic entry. Inhibiting WEE1 permits premature entry into M-phase and results in metaphase arrest [31]. This suggests that metaphase arrest inhibits HIV-1 spread. In contrast, CDK1 is required for progressing from G2 through M phase. Inhibiting CDK1 causes G2 arrest [102] and reduced viral spread. This suggests that the transition into and out of M-phase modulates viral spread. However, it should be noted that our assays have a relatively short incubation with the inhibitor of only 2 hours, which seems insufficient to arrest enough cells to mediate these effects solely through the cell cycle. Notably, both WEE1 (Fig 4E) and CDK1 [103] directly or indirectly regulate the function of AURKB. AURKB activity decreased after co-culture of Env+ and CD4+ cells and inhibiting AURKB activity greatly enhanced cell to cell spread (Figs 4B, 4C and 5). Because of the close interplay between these three proteins, and the unique phenotype of inhibition leading to enhanced viral spread, we focused on characterizing the role of AURKB during HIV-1 spread.

AURKB’s role in HIV-1 spread was confirmed by using two independent AURKB inhibitors (Figs 4 and 5) and correlating with the negative effects of WEE1 inhibition (Fig 4B and 4C), which stimulated AURKB activity (Fig 4E). AURKB regulates multiple steps in mitotic chromosomal segregation and cytokinesis [32]. Previously, HIV-1-infected T cells were shown to have elevated AURKA and AURKB activity relative to uninfected cells [23,73,74,104]. This appears due to Vif degradation of regulatory subunits of PP2A phosphatases that modulate Aurora kinase activity, rather than to direct Vif effects on Aurora kinases [105,106].

How this early increase in AURKA and AURKB activity may contribute to HIV-1 infection remains to be determined. In one recent study, productive HIV-1 cell-free infection of primary T cells was inhibited by a selective AURKA inhibitor but not by a selective AURKB inhibitor, barasertib [104]. Our experiments agree that barasertib treatment of target cells does not inhibit cell-free virion spread (Fig 4D). However, further comparisons are limited because these studies differed from ours (a) in being focused on cell-free infections, rather than on interactions between barasertib-sensitive HIV-1-infected producer cells and target cells, and (b) in using VSV-G-pseudotyped virions, thus preventing induction or detection of any of the HIV-1 Env-mediated effects crucial to our studies.

**Fig 11:**
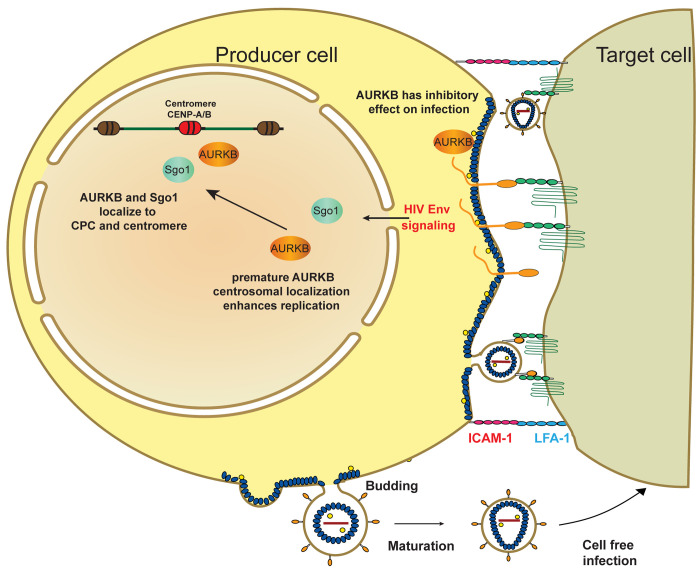
AURKB regulation of HIV spread through virological synapse requires the C-terminal domain of Env. Schematic of HIV-1 virological synapse. Cytoplasmic AURKB exerts an unknown negative effect on HIV-1 spread through the virological synapse which reduces the fusion activity of the HIV-1 Env protein. HIV-1 overcomes this through the CTD of HIV-1 Env which induces premature nuclear localization of AURKB to the CPC.

Nevertheless, our kinase activity analysis revealed that co-culturing infected cells expressing HIV-1 Env with uninfected cells induced a dramatic decrease in AURKB kinase activity in HIV-1 producer cells within an hour of co-culture (Fig 3C), and two AURKB-specific inhibitors each markedly stimulated infected / uninfected cell interaction and HIV-1 spread (Figs 4 and 5). Thus, while HIV-1 infection increases AURKA and AURKB levels in newly infected cells, later cell-cell interactions and Env signaling appear to function to counter undesirable AURKB activity at the plasma membrane that would otherwise inhibit Env fusogenicity and HIV-1 spread (Fig 11).

Upon AURKB inhibition we observed extended cell-cell contacts and an increase in syncytia, demonstrating alterations in the fusion activity of Env (Fig 5). Further studies might explore how AURKB inhibition mediates increased Env fusogenicity and whether this involves changes in the state of Env, the membrane, underlying actin, or other factors. Notably, Env was the only HIV-1 protein required for AURKB inhibition-induced syncytia formation (Fig 5H) and this effect was mediated through the Env CTD (Fig 6D). Interestingly, while HIV-1 is a highly fusogenic virus with significant cell-to-cell transfer, in most cases few syncytia form relative to the number of VSs [5,6,107]. Indeed, large syncytia are associated with rapidly dying cells [108,109] and only small (5 nuclei or less) syncytia may transmit virus in vivo [110,111]. These observations and our data suggest that the fusion process and syncytia formation are tightly regulated.

AURKB activity is primarily regulated by subcellular localization [32]. We showed that AURKB relocalized to or immediately next to centrosomes after producer cell–target cell interaction (Figs 6–8). This effect was entirely dependent on HIV-1 Env:CD4 engagement (Fig 7), was independent of co-receptor usage (Fig 7A) and required the cytoplasmic tail domain (CTD) of Env in both Jurkat (Figs 7–9) and primary T cells (Fig 10). Finally, we directly scored the effects of Env:CD4 engagement on cell cycle progression and AURKB relocalization, finding enhanced AURKB localization to centrosomes with no changes in the cell cycle (Fig 9). Interestingly, this relocalization was specific, considering that the majority of centromere and CPC components we examined were not relocalized by Env:CD4 interaction. Moreover, the CPC-regulating protein SGO1 also relocalized after Env:CD4 interaction, either through a similar signaling pathway or through interaction with AURKB (Fig 8). Alternatively, SGO1, whose role is to prevent and regulate cohesin phosphorylation [112,113], could be responding to the increase in premature AURKB at the centromere. Of the three AURK family members, AURKA was not relocalized (Fig 8), while the more closely related AURKC [32] relocalized like AURKB after Env:CD4 interaction (Fig 8).

While the vast majority of AURKB studies have largely focused on its roles in specific steps of mitosis, our findings suggest that AURKB has significant effects in interphase that negatively affect HIV-1 cell-cell transfer beyond its involvement in mitotic regulation. Intriguingly, AURKB has been characterized as localizing to the plasma membrane during interphase [114]. This is consistent with the role of AURKB during cytokinesis. Membrane scission during cytokinesis is mediated by AURKB interactions with the cellular endosomal sorting complex required for transport (ESCRT) [115,116], which functions in multiple membrane scission events during cytokinesis [117–120] as well as HIV-1 release from the host plasma membrane [121–123]. Thus, AURKB- competition for or regulation of the ESCRT-III machinery essential for virion release might contribute to the observed repression of AURKB activity when HIV-1-infected cells contact uninfected target cells, and the stimulation of HIV-1 transmission upon AURKB chemical inhibition.

Alternatively, during cytokinesis, AURKB is involved in reorganizing the actin networks at the cleavage furrow [124,125]. VS formation may require localized reorganization of cortical actin that may be regulated by AURKB at the plasma membrane and HIV-1 needs to remove the inhibitory effect of AURKB for efficient cell to cell spread. This is potentially consistent with the observation that AURKB inhibition primarily increased spread though enhanced Env fusogenicity (Figs 5 and 6D). It has long been unclear what keeps VSs from expanding into elongated contacts or cellular fusion. Since we observed that global chemical inhibition of AURKB activity expanded cell-cell contacts and cellular fusion (Fig 5), regulation of AURKB might need to be highly localized to the immediate vicinity of Env:CD4 contacts to support Env fusogenicity while preventing excessive membrane fusion.

Recently, multiple viruses from diverse families including Epstein Barr virus (EBV) and Kaposi’s Sarcoma virus (KSHV), hepatitis C virus (HCV) and dengue virus were shown to be affected by or to modulate AURKB activity [126–129]. Of particular note, AURKB is critical for cytoplasmic assembly and plasma membrane budding of dengue virus [129]. These results are consistent with our observation that an unknown cytoplasmic role of interphase AURKB may regulate membrane function. AURKB inhibition may prove to have applications in targeting multiple viral families.

Interestingly, AURKB inhibitors are currently being investigated as anti-cancer drugs. Our data suggest they may be counter-indicated for HIV-positive cancer patients. Further, they suggest that AURKB plays critical roles in cellular pathways outside of cell division. In conclusion, by using novel cell labeling/TMT mass spectrometry approaches, we demonstrated that on contacting uninfected cells, HIV-1-infected cells undergo rapid and expanding signaling changes to facilitate viral spread to uninfected cells. This includes the premature relocalization of AURKB to centrosomes which shows a previously unknown function of AURKB at the plasma membrane regulating HIV Env fusogenicity. These approaches are widely applicable to studying other processes involving mixed cell populations and should offer new insights into how pathogens and cells modulate the cellular environment through cell to cell signaling.

## Supporting information

S1 Fig Western blot of cell mixing conditions. (A) Jurkat producer cells and uninfected SupT1 target cells were mixed for 0, 5 and 60 minutes. Cells were lysed, separated by SDS-PAGE and immunoblotted with the indicated antibodies. Consistent with previous studies and our mass spec results, no changes were observed in viral Env or gag or in total cellular actin or MAPK14. Phospho-MAPK14 was unchanged 5 min after mixing and decreased by 60 min. In contrast, p-AKT increased at 5 and 60 min post mixing. (B) Quantitation of p-AKT and p-MAPK14. The data shown are the average mean values obtained in an experiment performed with quadruplicate samples and are representative of three independent experiments. Error bars indicate the standard deviation of the data. P-values were calculated using a standard Student’s t-test.
(EPS)

S2 Fig Volcano plots. Fold changes and statistical significance of the (A) protein abundance changes at 5 min, (B) protein abundance changes at 60 min, (C) protein phosphorylation changes at 5 min, and (D) protein phosphorylation changes at 60 min. Statistical test results from the same time point and different multiplexes are displayed in the same volcano plot. Flat portions in some q-value distributions are due to the q-value being defined as the minimum false discovery rate that can be achieved when including a phosphopeptide or protein as significant. Because q-values depend on the number of statistical tests performed and the number of peptides quantified is different in each multiplex, the range of -log10 q-values differs for each multiplex.
(EPS)

S3 Fig Schematic of computational approach for network analysis and kinase enrichment. Data from three multiplexes were quantile normalized and fold changes were calculated. These data were used for kinase analysis. After statistical analysis, network analysis was performed and Gene Ontology enrichment was determined based on the nodes in the aggregated networks.
(EPS)

S4 Fig Protein subnetworks in HIV-1 producer cells after 5 minutes of co-culture with uninfected cells. Prize-Collecting Steiner Forest (PCSF) analysis was used to generate subnetworks from proteins with significant changes after 5 minutes of co-culture. The protein-protein interaction subnetworks created using the PCSF algorithm from significantly differentiated proteins and phosphopeptides. The subnetworks depict all edges of 75% or greater confidence. Vertex color of the elliptical vertices represents the magnitude of the log-transformed q-values, which were used as protein prizes. Steiner nodes, vertices that were not significantly changed between time points but were included as important connective proteins by the PCSF algorithm, are shown as rectangles.
(PDF)

S5 Fig Protein subnetworks in HIV-1 producer cells after 60 minutes of co-culture with uninfected cells. Prize-Collecting Steiner Forest (PCSF) analysis was used to generate subnetworks from proteins with significant changes after 60 minutes of co-culture. The protein-protein interaction subnetworks created using the PCSF algorithm from significantly differentiated proteins and phosphopeptides. The subnetworks depict all edges of 90% or greater confidence. Vertex color of the elliptical vertices represents the magnitude of the log-transformed q-values, which were used as protein prizes. Steiner nodes, vertices that were not significantly changed between time points but were included as important connective proteins by the PCSF algorithm, are shown as rectangles.
(PDF)

S6 Fig Decision tree used to identify proteins involved in HIV cell to cell spread. Initial screening was performed using producer cell mixing with TZM-BL target cells (Fig 4A). Potential hits were confirmed by further analysis as shown. Compounds were rejected if either producer or target cells viability was negatively affected by chemical treatment (Fig 4B). Next, potential hits were rejected if chemical treatment inhibited cell free virion infection of target cells (Fig 4D). Finally, if inhibitors enhanced syncytia formation, they were classified as a specific class of hit that affected the fusogenic activity of Env and selected for further study (Fig 5).
(EPS)

S1 Table Producer cell Proteins identified after VS formation. Proteins identified by protein group, UniProt ID, and representative name. Columns show log2 fold changes in total protein level and q-values in experiments 1 and 2 at 5 and 60 minutes relative to 0 min controls. True in the Significant column means the protein met criteria of a q-value ≤ 0.1 and a fold change ≥ 1.5 in magnitude in at least one experiment and time point.
(XLSX)

S2 Table Producer cell phosphopeptides identified after VS formation. Phosphopeptides identified by protein group, UniProt ID, representative name, and phosphorylation isoform. Columns show log2 fold changes and q-values in phosphopeptide level in experiments 1 and 2 at 5 and 60 minutes relative to 0 min controls. True in the Significant column means the phosphopeptide met criteria of a q-value ≤ 0.1 and a fold change ≥ 1.5 in magnitude in at least one experiment and time point.
(XLSX)

S3 Table Kinase activity. Kinase activities from the phosphorylation log2 fold changes at 5 min using PhosFate Profiler. PhosFate Profiler computes an enrichment score for kinases using the Kolmogorov–Smirnov statistical test, a p-value, and a kinase activity, where a positive score indicates an increase and a negative score a decrease in kinase activity.
(XLSX)
