# Supplementary figures and images for: HIV-1 virological synapse formation enhances infection spread by dysregulating Aurora Kinase B
Source: PLoS Pathog. 2023 Jul 17;19(7):e1011492. doi: 10.1371/journal.ppat.1011492 (PMC10374047; doi:10.1371/journal.ppat.1011492)

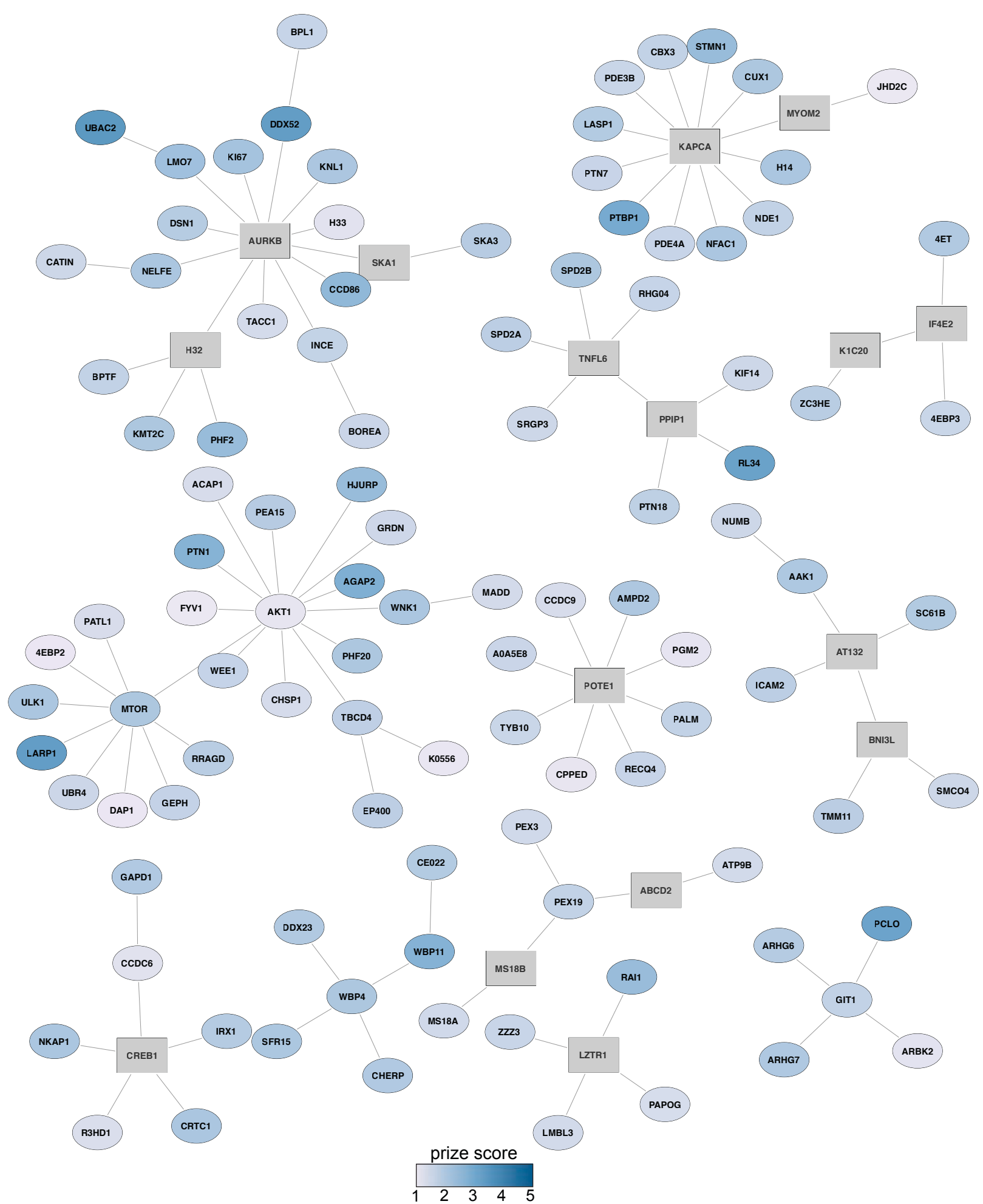

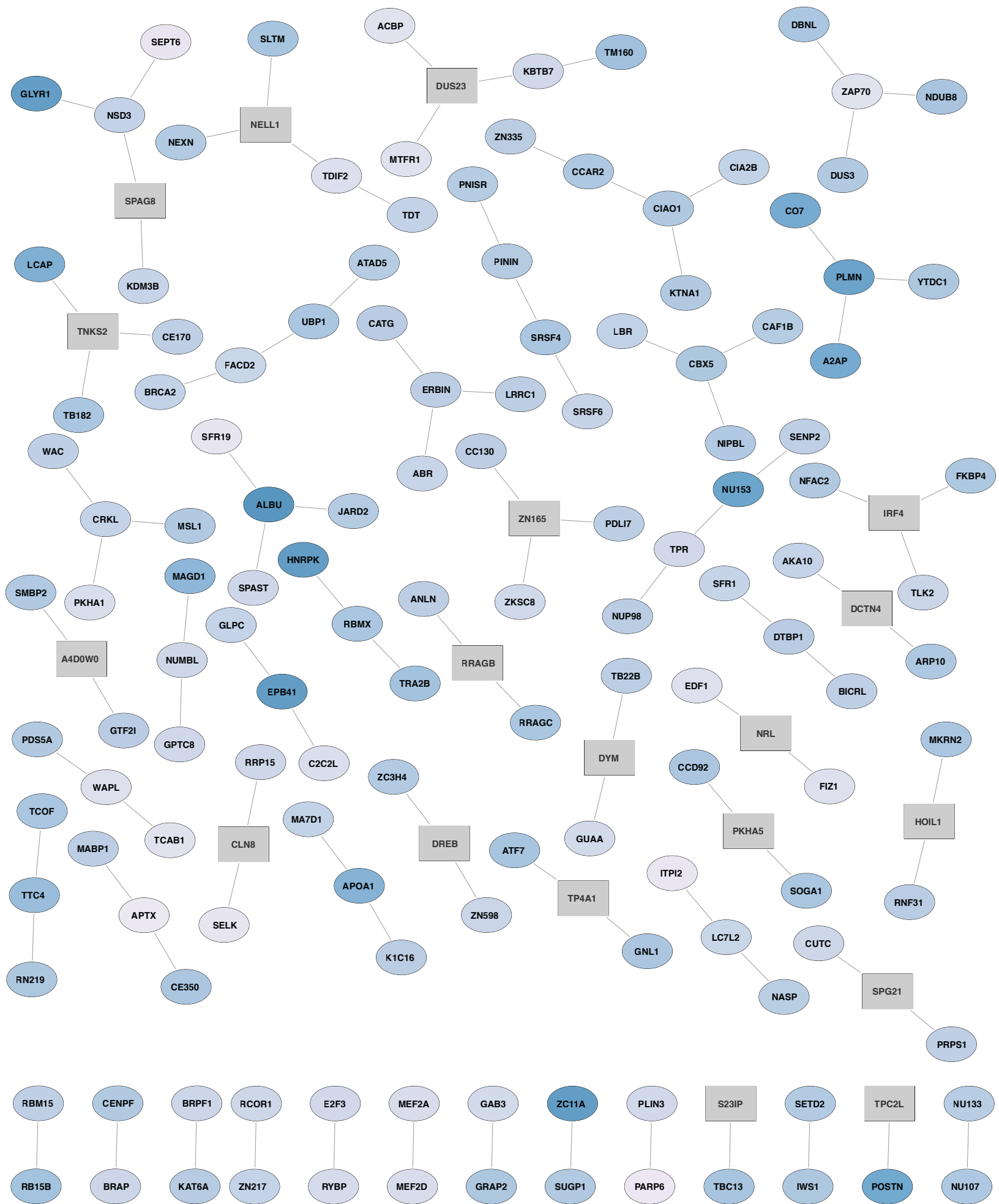

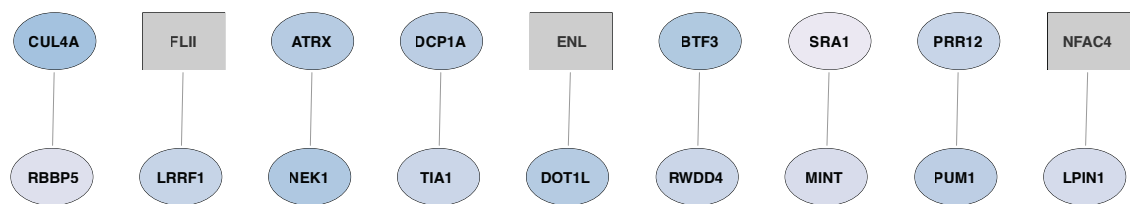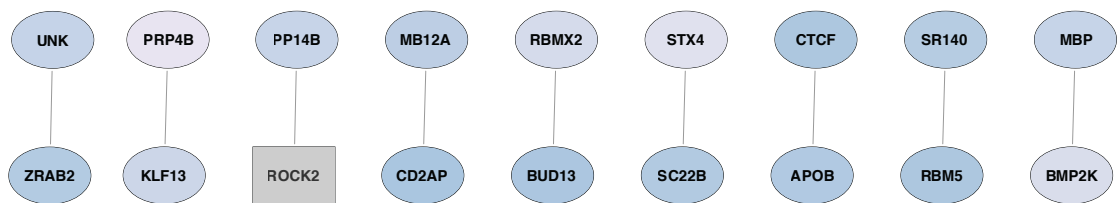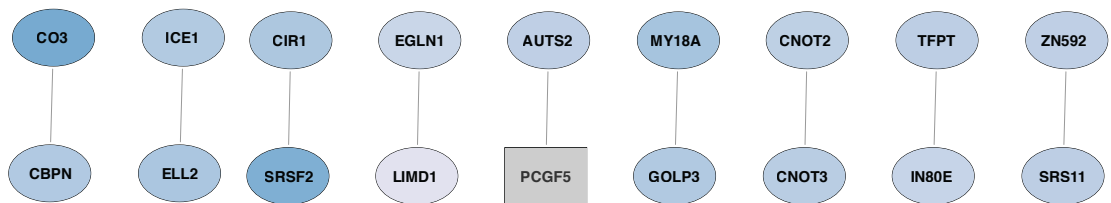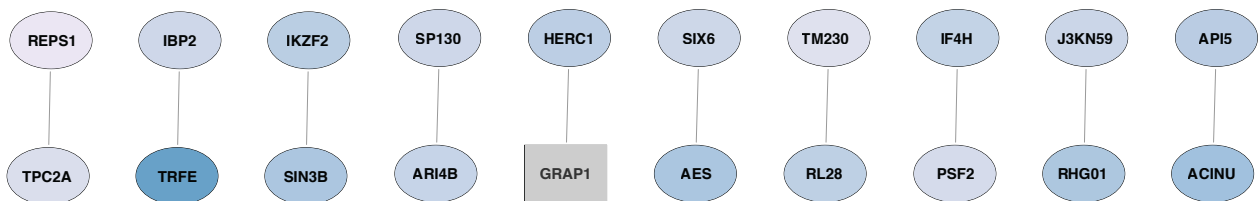

Supplement: S4 Fig — Prize-Collecting Steiner Forest (PCSF) analysis was used to generate subnetworks from proteins with significant changes after 5 minutes of co-culture. The protein-protein interaction subnetworks created using the PCSF algorithm from significantly differentiated proteins and phosphopeptides. The subnetworks depict all edges of 75% or greater confidence. Vertex color of the elliptical vertices represents the magnitude of the log-transformed q-values, which were used as protein prizes. Steiner nodes, vertices that were not significantly changed between time points but were included as important connective proteins by the PCSF algorithm, are shown as rectangles. (PDF) [file ppat.1011492.s004.pdf]

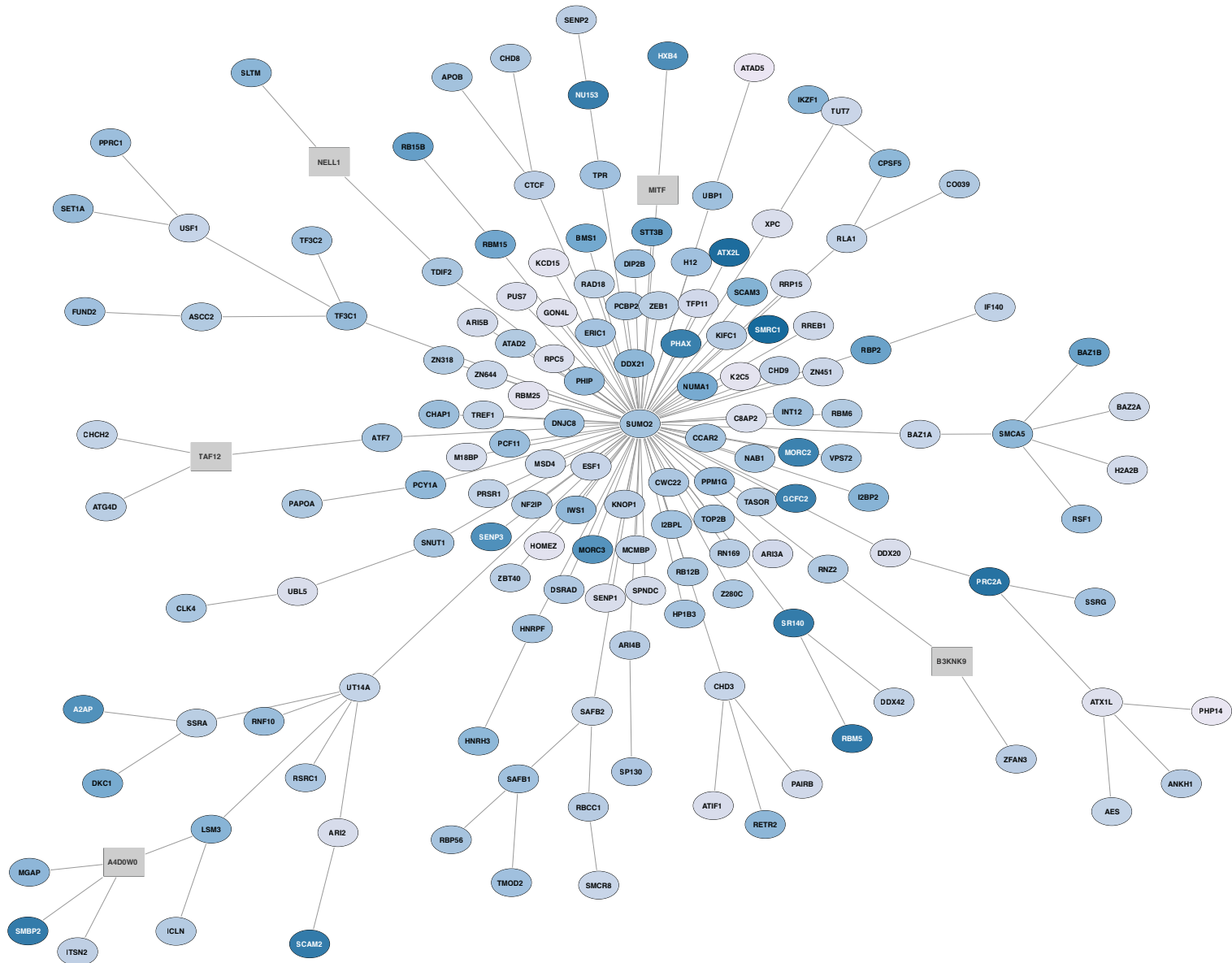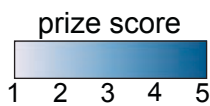

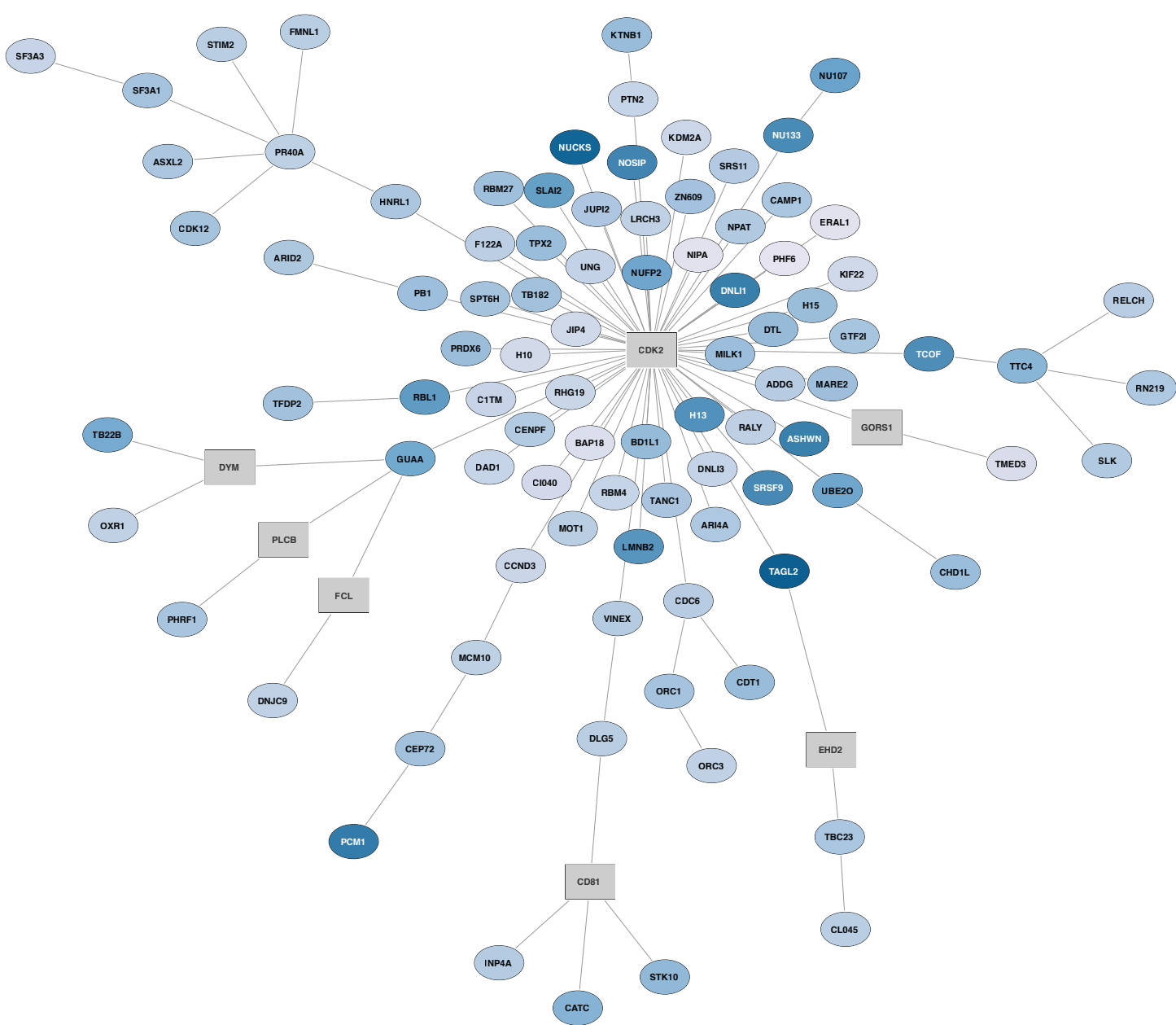

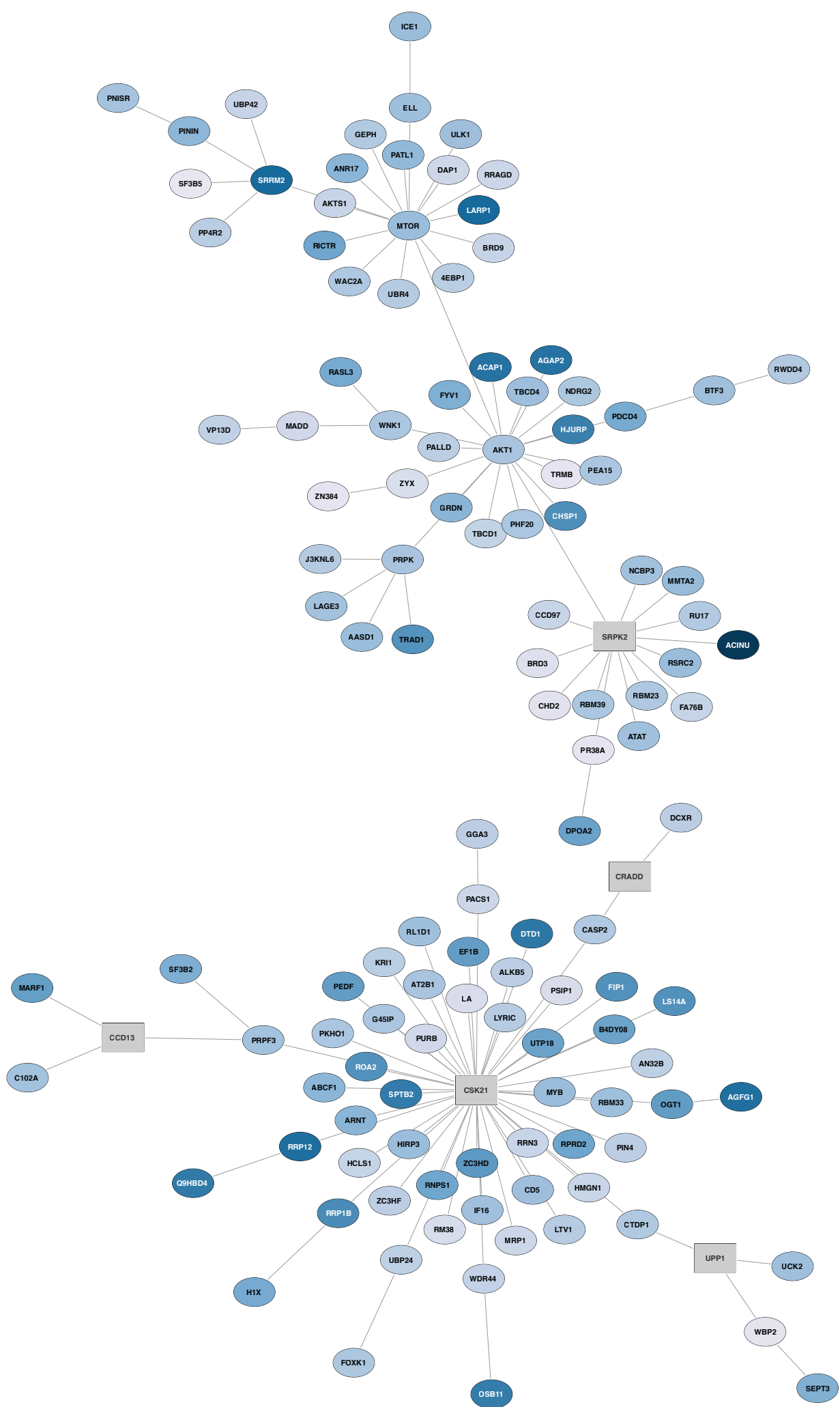

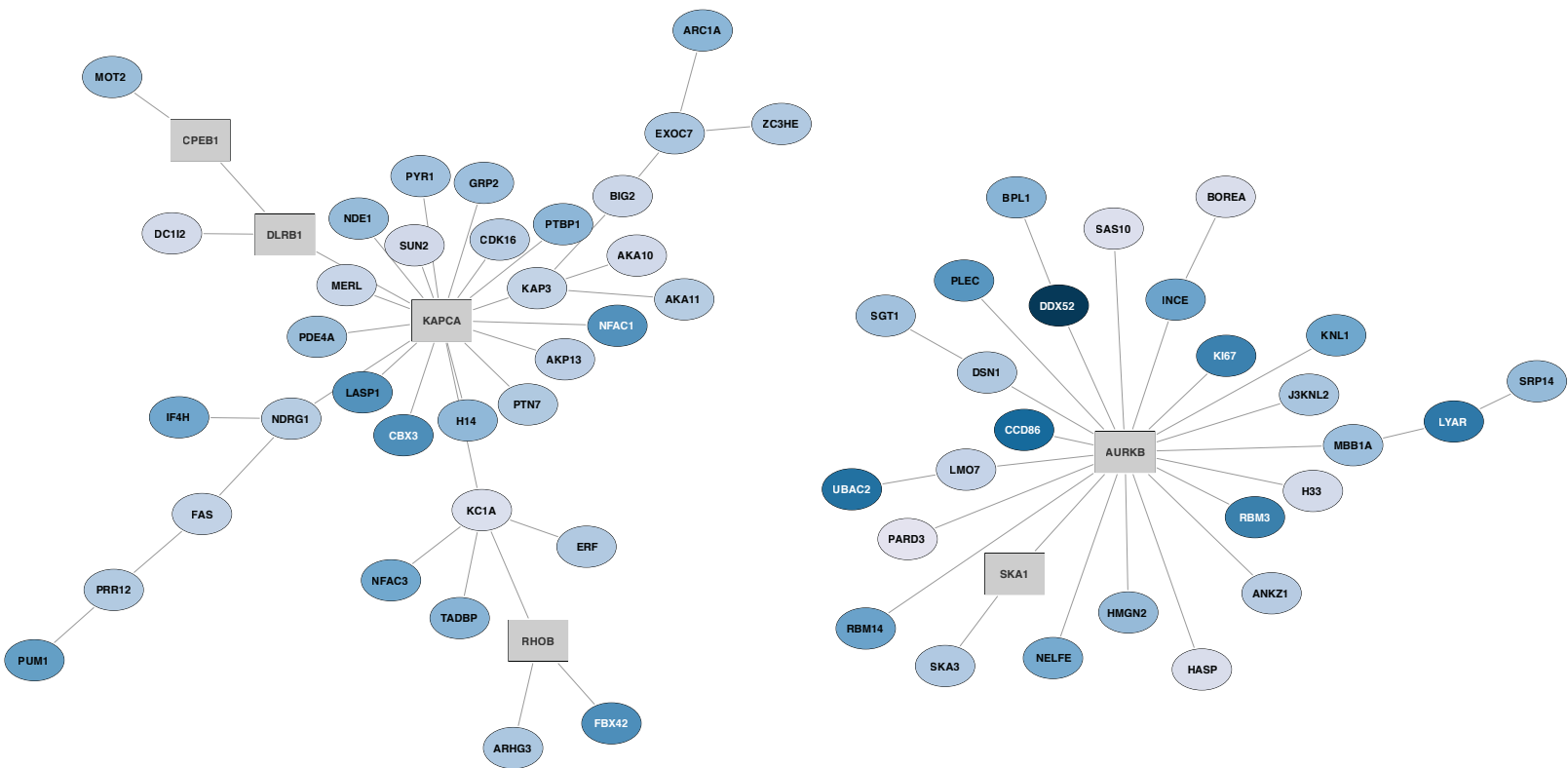

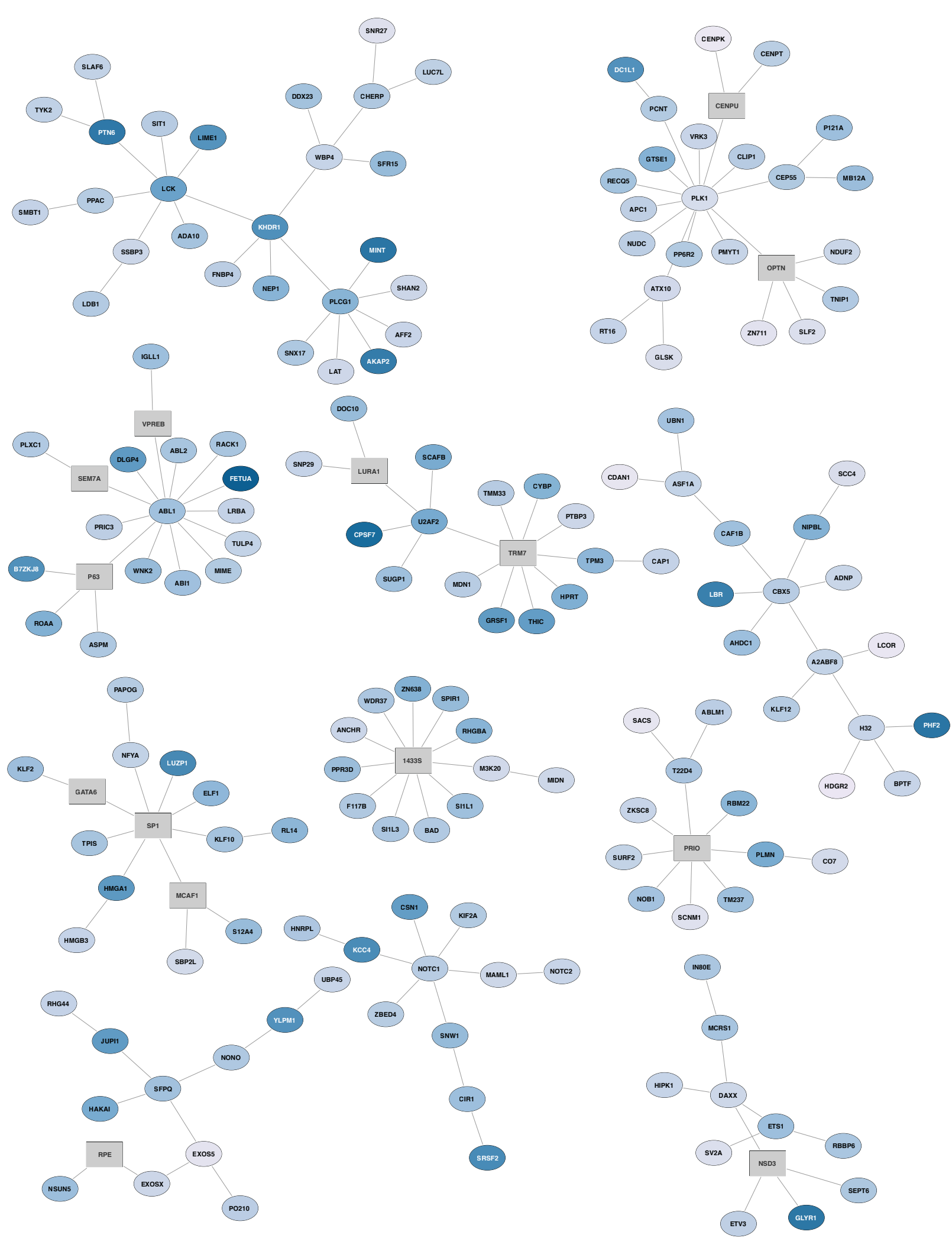

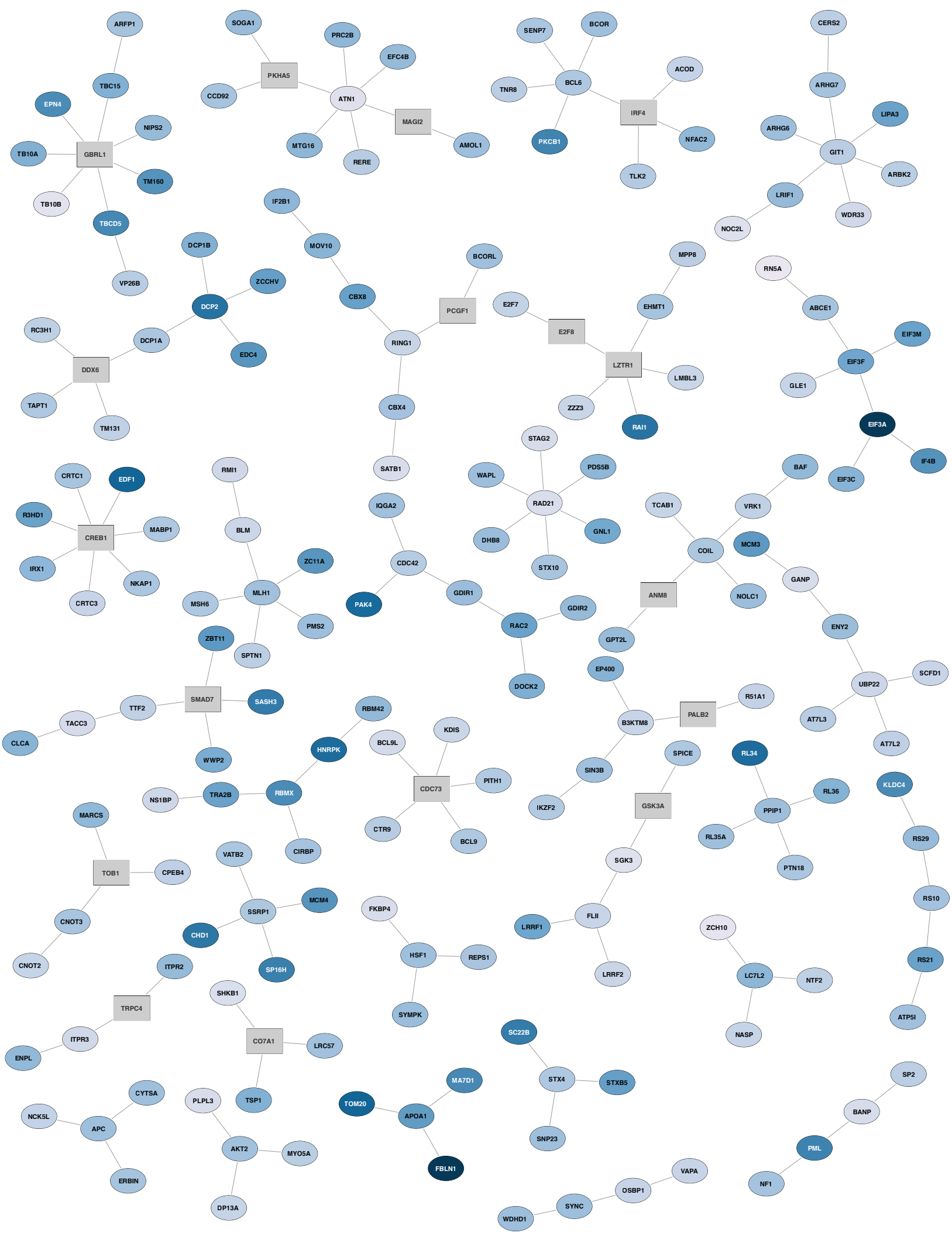

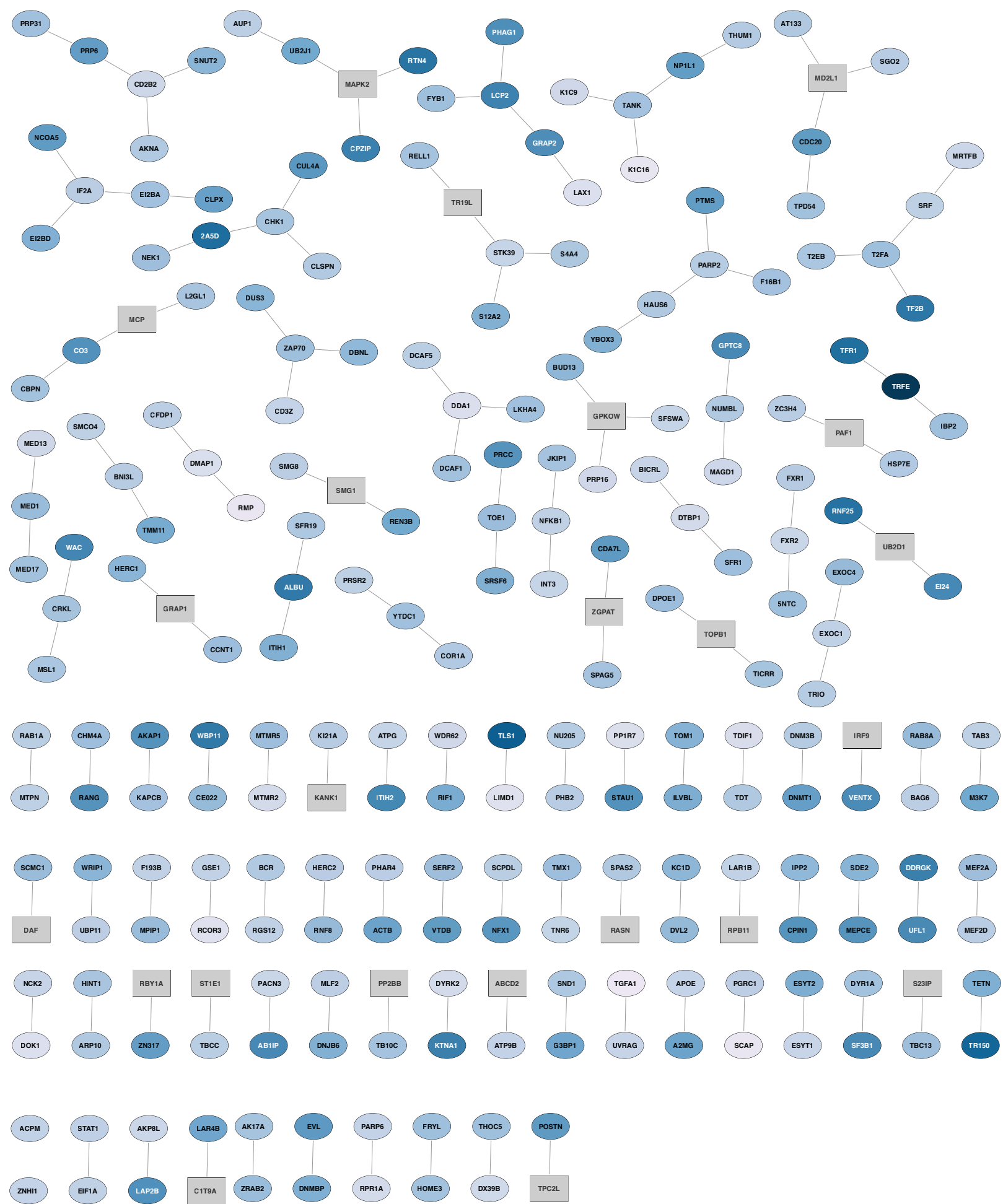

Supplement: S5 Fig — Prize-Collecting Steiner Forest (PCSF) analysis was used to generate subnetworks from proteins with significant changes after 60 minutes of co-culture. The protein-protein interaction subnetworks created using the PCSF algorithm from significantly differentiated proteins and phosphopeptides. The subnetworks depict all edges of 90% or greater confidence. Vertex color of the elliptical vertices represents the magnitude of the log-transformed q-values, which were used as protein prizes. Steiner nodes, vertices that were not significantly changed between time points but were included as important connective proteins by the PCSF algorithm, are shown as rectangles. (PDF) [file ppat.1011492.s005.pdf]
